# Total Synthesis of Decahydroquinoline Poison Frog Alkaloids ent-*cis*-195A and *cis*-211A

**DOI:** 10.3390/molecules26247529

**Published:** 2021-12-12

**Authors:** Takuya Okada, Naizhen Wu, Katsuki Takashima, Jungoh Ishimura, Hiroyuki Morita, Takuya Ito, Takeshi Kodama, Yuhei Yamasaki, Shin-ichi Akanuma, Yoshiyuki Kubo, Ken-ichi Hosoya, Hiroshi Tsuneki, Tsutomu Wada, Toshiyasu Sasaoka, Takahiro Shimizu, Hideki Sakai, Linda P. Dwoskin, Syed R. Hussaini, Ralph A. Saporito, Naoki Toyooka

**Affiliations:** 1 Graduate School of Innovative Life Science, University of Toyama, 3190 Gofuku, Toyama 930-8555, Japan; 2 Graduate School of Medicine and Pharmaceutical Sciences, University of Toyama, 2630 Sugitani, Toyama 930-0194, Japan; wnz199242@gmail.com (N.W.); s1860342@ems.u-toyama.ac.jp (Y.Y.); akanumas@pha.u-toyama.ac.jp (S.-i.A.); kubo.yoshiyuki.jf@teikyou-u.ac.jp (Y.K.); hosoyak@pha.u-toyama.ac.jp (K.-i.H.); htsuneki@pha.u-toyama.ac.jp (H.T.); twada@pha.u-toyama.ac.jp (T.W.); tsasaoka@pha.u-toyama.ac.jp (T.S.); takshimi@pha.u-toyama.ac.jp (T.S.); sakaih@pha.u-toyama.ac.jp (H.S.); 3 Graduate School of Science and Engineering, University of Toyama, 3190 Gofuku, Toyama 930-8555, Japan; takashima@phar.kindai.ac.jp (K.T.); junirad@gmail.com (J.I.); 4 Institute of Natural Medicine, University of Toyama, 2630 Sugitani, Toyama 930-0194, Japan; hmorita@inm.u-toyama.ac.jp (H.M.); itoutaku@osaka-ohtani.ac.jp (T.I.); tkodama@inm.u-toyama.ac.jp (T.K.); 5 Faculty of Pharmacy, Osaka Ohtani University, Tondabayashi, Osaka 584-8540, Japan; 6 Department of Pharmaceutical Sciences, College of Pharmacy, University of Kentucky, Lexington, KY 40536, USA; ldwoskin@email.uky.edu; 7 Department of Chemistry and Biochemistry, The University of Tulsa, 800 S. Tucker Dr., Tulsa, OK 74104, USA; syed-hussaini@utulsa.edu; 8 Department of Biology, John Carroll University, University Heights, OH 44118, USA; ralph.saporito@gmail.com

**Keywords:** decahydroquinoline, poison frog alkaloid, *cis*-**195A**, *cis*-**211A**

## Abstract

The total synthesis of two decahydroquinoline poison frog alkaloids ent-*cis*-**195A** and *cis*-**211A** were achieved in 16 steps (38% overall yield) and 19 steps (31% overall yield), respectively, starting from known compound **1**. Both alkaloids were synthesized from the common key intermediate **11** in a divergent fashion, and the absolute stereochemistry of natural *cis*-**211A** was determined to be 2*R*, 4a*R*, 5*R*, 6*S*, and 8a*S*. Interestingly, the absolute configuration of the parent decahydroquinoline nuclei of *cis*-**211A** was the mirror image of that of *cis*-**195A**, although both alkaloids were isolated from the same poison frog species, *Oophaga* (*Dendrobates*) *pumilio*, from Panama.

## 1. Introduction

**Figure 1:**
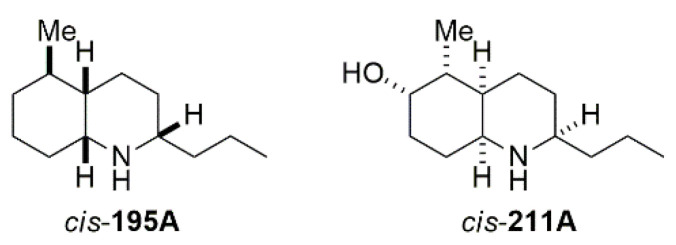
Structures of *cis*-**195A** and *cis*-**211A**.

The skin extracts of Neotropical poison frogs contain a variety of lipophilic alkaloids, and over 800 alkaloids have been isolated or detected to date [1]. Many of these alkaloids show remarkable biological activities on the nervous system such as nicotinic acetylcholine receptors [2,3]. Methods for chemical synthesis of poison frog alkaloids are needed to investigate the biological activities of poison frog alkaloids, as only minute amounts of natural alkaloids can be obtained from skin extracts [1]. Decahydroquinolines are a relatively large subgroup of poison frog alkaloids, and over 50 types have been detected. Among them, the alkaloid *cis*-**195A** is the parent member of this class that was originally isolated from a Panamanian population of *Oophaga* (*Dendrobates*) *pumilio* in 1969 [4]. The structure and absolute configuration of *cis*-**195A** were determined by X-ray crystallography, and several total syntheses have also been reported for this compound [5,6,7,8,9,10,11,12,13,14,15,16,17,18,19,20,21,22]. The alkaloid *cis*-**211A** was isolated from skin extracts of the same species of poison frog in 1987 [23]. However, no total synthesis of this alkaloid has been reported, and its absolute configuration remains unknown to date (Figure 1). As part of a program directed at studying the synthesis of poison frog alkaloids [24,25,26,27,28,29,30,31,32,33,34,35,36,37,38], herein, we report the total synthesis of ent-*cis*-**195A** and *cis*-**211A**. Both syntheses proceed via the common and key intermediate **11**. The synthesis of *cis*-**211A** also enabled the determination of its absolute configuration.

## 2. Results and Discussion

**Scheme 1:**
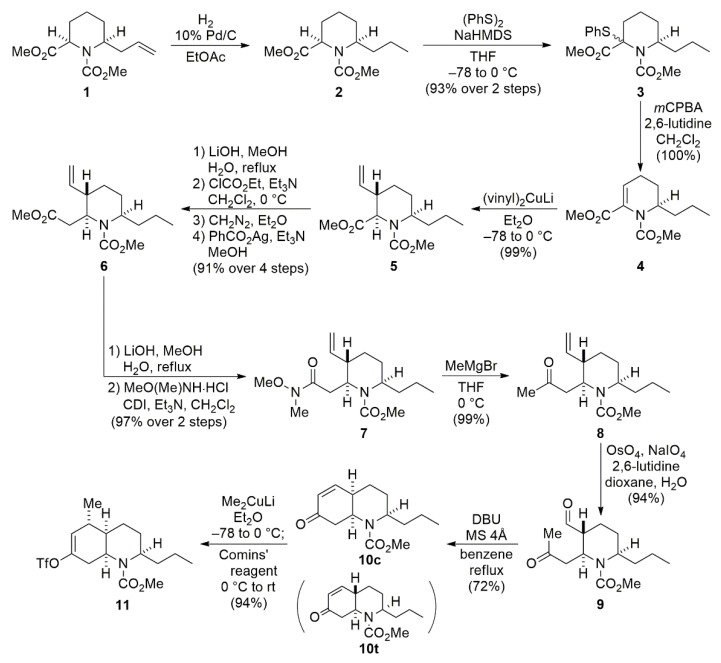
Synthesis of the common and key intermediate enoltriflate **11**.

**Figure 2:**
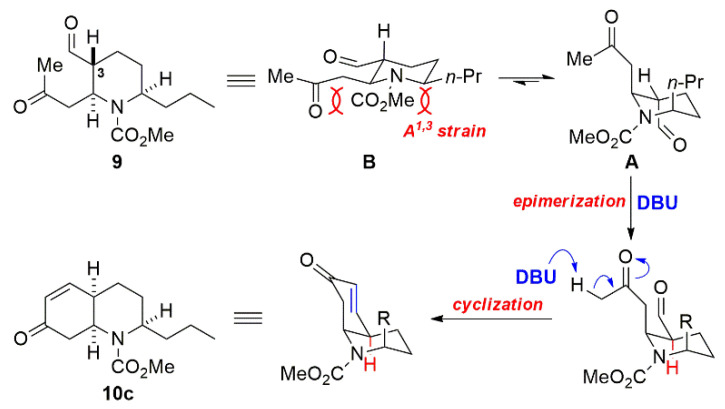
Reaction mechanism of the cyclization with epimerization of **9**.

Hydrogenation of known allyl derivative **1** [39] provided the ester **2**, which was converted to enaminoester **4** via thiophenyl derivative **3**. The Michael-type conjugate addition reaction to **4** [25] gave adduct **5** as a single isomer in excellent yield. The ester moiety of **5** was elongated by the Arndt–Eistert reaction sequence to afford the homologated ester **6**, which was transformed into the methyl ketone **8** via the corresponding Weinreb amide **7**. Lemieux–Johnson oxidation of **8** provided the aldehyde **9**, which was subjected to cyclization by treatment with DBU in refluxing benzene to yield the *cis*-fused enone **10c** [25] without generating the *trans*-fused enone **10t**. Selective formation of **10c** was explained by the preferential formation of conformer **A** in the starting material **9** owing to the *A*^1,3^ strain. Thus, epimerization at the 3-position of **9** occurred first, and then cyclization proceeded to provide the enone **10c** as shown in Figure 2. With enone **10c** in hand, the stage was set for the divergent synthesis of ent-*cis*-**195A** and *cis*-**211A**. The conjugate addition reaction to **10c** with Me_2_CuLi followed by treatment of the resulting enolate with Comins’ triflating agent [40] afforded the common and key intermediate **11** (Scheme 1).

**Scheme 2:**
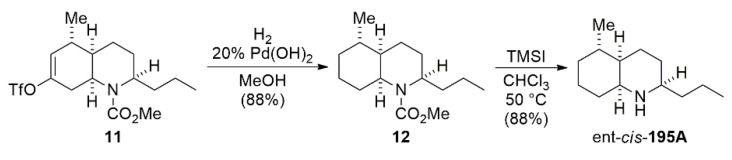
Total synthesis of ent-*cis*-**195A**.

Finally, global hydrogenation of **11** and deprotection of the methyl carbamate moiety in **12** using TMSI in CHCl_3_ at 50 °C provided ent-*cis*-**195A**, as shown in Scheme 2. The ^1^H and ^13^C-NMR spectra of synthetic ent-*cis*-**195A** were in good agreement with those reported in the literature [22].

**Scheme 3:**
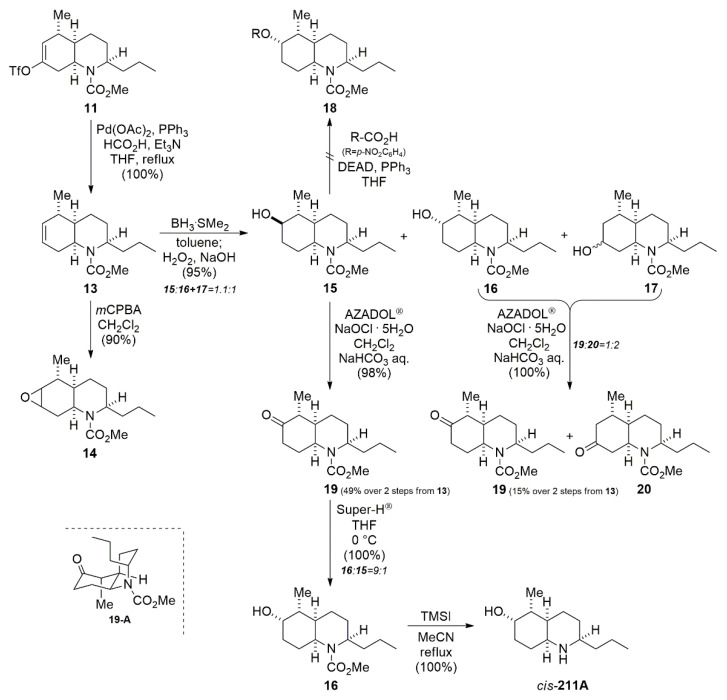
Total synthesis of *cis*-**211A**.

The enol triflate **11** was converted to olefin **13** by palladium-catalyzed reduction. Epoxidation of **13** by *m*CPBA proceeded smoothly to give the epoxide **14**, unfortunately, as a 1:1 mixture of epoxide **14**. Hydroboration of **13** using BH_3_-SMe_2_ in toluene provided the alcohol **15** and the mixture of alcohols **16** and **17**. The structure of **15** was determined by NOESY. The NOESY experiments of **15** revealed a syn relationship between the methyl group at C-5 and H-6 based on the NOESY correlations from H_3_-5 to H-6. However, the separation of **16** and **17** was difficult at this stage. For completion of the synthesis of *cis*-**211A**, inversion of the hydroxyl group in **15** was necessary. For this purpose, we subjected **15** to the Mitsunobu reaction; however, all attempts failed and resulted in the recovery of **15**. Next, we examined hydroxyl inversion of **15** via ketone **19**. Any oxidations of **15** using Swern, a SO_3_-pyridine complex, PCC, PDC, DMP, or TPAP were not successful, and the starting material was recovered. Only oxidation using AZADOL^®^ [41] proceeded smoothly to yield the desired ketone **19** in good yield. In addition, the AZADOL^®^ oxidation of the mixture of **16** and **17** afforded the ketones **19** and **20** in 34% and 66% yield, respectively, which could be easily separated by column chromatography. Thus, we succeeded to obtain the ketone **19** from **13** in 64% (49% + 15%) overall yield, as shown in Scheme 3. The conformation of ketone **19** is depicted in **19-A**. The reduction of **19** from the concave face was needed to obtain the desired alcohol **16**. We expected that the reduction of **19** would proceed from the concave face, as shown in **19-A**, because of the steric hindrance of the α-axial methyl group. However, the use of a small reducing agent like NaBH_4_ or LiAlH_4_ reduced **19** from the convex face to afford **15** as the sole product. To secure the reduction from the concave face, we tried the large reducing agent L-Selectride^®^; however, the reduction did not proceed, and only ketone **19** was recovered. Fortunately, Super-Hydride^®^, a moderately sized reducing agent, was the best match for this substrate, and the reduction proceeded from the concave face to provide the desired alcohol **16** as the major product (**16**:**15** = 9:1). The final deprotection of the urethane moiety in **16** was also troublesome. First, we applied the same reaction conditions used for ent-*cis*-**195A** (TMSI in refluxing CHCl_3_) to cleave the methyl carbamate; however, the reaction did not proceed. Then, other reaction conditions, such as the use of *n*-PrSLi/HMPA or KOH/*i*-PrOH in a sealed tube at 130 °C, resulted in the recovery of the starting material. Finally, we used TMSI in refluxing MeCN, and the reaction proceeded cleanly to yield *cis*-**211A**, as shown in Scheme 3.

The ^1^H- and ^13^C-NMR spectra of synthetic *cis*-**211A** were in good agreement with the reported values [23]. The absolute stereochemistry of natural *cis*-**211A** was determined unambiguously by the present synthesis to be 2*R*, 4a*R*, 5*R*, 6*S*, and 8a*S* by comparison of the optical rotation of synthetic *cis*-**211A** ([α]_D_^25^ −11.5 (*c* 0.2, CHCl_3_)) with the reported value ([α]_D_ −11.7 (*c* 1.0, CHCl_3_)) [23]. Interestingly, *cis*-**195A** and *cis*-**211A** were both isolated from the same poison frog, *Oophaga* (*Dendrobates*) *pumilio* (Dendrobatidae) from Panama; however, the absolute stereochemistry of the parent decahydroquinoline nuclei of *cis*-**195A** is opposite to that of *cis*-**211A**. The NMR spectra (^1^H-NMR, ^13^C-NMR) of all synthesized compounds are listed in Supplementary Materials.

**Scheme 4:**
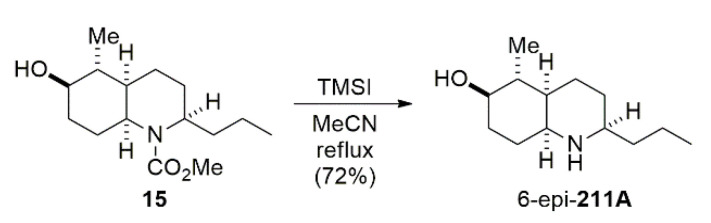
Synthesis of 6-epi-**211A**.

To further investigate the effect of the stereochemistry of the hydroxyl group at the 6-position on the inhibitory activity against nicotinic acetylcholine (ACh) receptors, we also synthesized 6-epi-**211A** by deprotection of the methoxycarbonyl group in **15**, as shown in Scheme 4.

**Figure 3:**
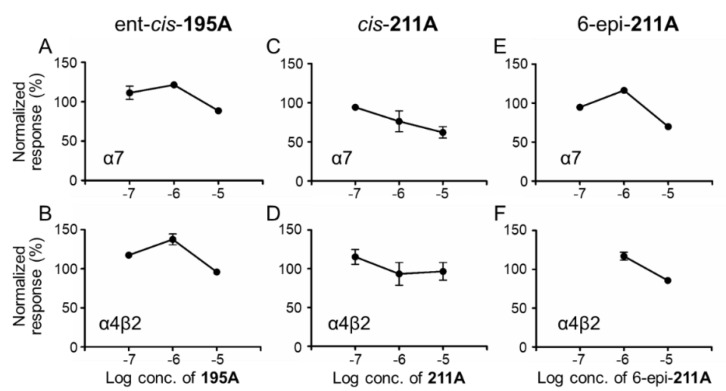
The effects of ent-*cis*-**195A**, *cis*-**211A**, and 6-epi-**211A** on α7- and α4β2-nicotinic acetylcholine receptors expressed in *Xenopus* oocytes. (**A**–**F**). Concentration–inhibition curves for ent-*cis*-**195A** (**A,B**), *cis*-**211A** (**C**,**D**), and 6-epi-**211A** (**E**,**F**) on α7- (**A**,**C**,**E**) and α4β2-nicotinic receptors (**B**,**D**,**F**). Current response to acetylcholine (ACh) in the presence of alkaloid was normalized to the current elicited by ACh alone in the same oocyte and averaged. Values represent the mean ± S.E.M. (**A**–**D**): *n* = 3–5, E: *n* = 3–4, F: *n* = 4–5.

Nicotinic ACh receptors are ligand-gated cation channels [42,43]. Homomeric α7- and heteromeric α4β2-pentamers are the major subtypes of nicotinic receptors found in the central nervous system [44]. It has been reported that (−)-*cis*-**195A**, a natural *cis*-decahydroquinoline alkaloid (formerly referred to as Pumiliotoxin C), blocks ganglionic nicotinic ACh receptors in pheochromocytoma PC12 cells [45] and that the synthetic analog (+)-*cis*-**195A** is more potent than (−)-*cis*-**195A** at inhibiting nicotinic receptor activity [3]. Here, we examined the effects of ent-*cis*-**195A**, *cis*-**211A**, and 6-epi-**211A** on α7- and α4β2-nicotinic ACh receptors ectopically expressed in *Xenopus* oocytes. If the criterion for partial inhibition by an alkaloid is defined as a ≥ 20% decrease in the peak amplitude of ACh-elicited currents, then ent-*cis*-**195A** (1–10 μM) showed no apparent inhibitory effects on α7- and α4β2-receptor-mediated currents (Figure 3A,B). *cis*-**211A** and 6-epi-**211A** at 10 μM partially inhibited α7-receptor-mediated currents by 38% and 31%, respectively, while both alkaloids at 10 μM showed negligible effects on α4β2-receptor-mediated currents (Figure 3C–F). Analysis of their structure–activity relationship suggested that the 6-hydroxy moiety of *cis*-**211A** and 6-epi-**211A** might contribute to the partial blockade of α7-nicotinic ACh receptors. The ligand-binding assays showed that none of these alkaloids affected [^3^H]nicotine and [^3^H]methyllycaconitine binding to rat whole brain membranes (data not shown). Therefore, *cis*-**211A** and 6-epi-**211A** were believed to act as noncompetitive blockers of α7-nicotinic receptors, although they were less potent and not as highly selective.

Neuronal nicotinic ACh receptors play a role in cerebral and retinal physiology. The blood–brain barrier (BBB) and inner blood–retinal barrier (BRB) directly segregate the brain and retina, respectively, from the circulating blood. It has been reported that putative nicotine- and verapamil-sensitive cationic drug transport systems at the BBB and inner BRB, respectively, are involved in the facilitative distribution of their substrates to the central nervous system [46,47]. To evaluate the recognition of ent-*cis*-**195A** and *cis*-**211A** as substrates for these cationic drug transport systems, we performed an inhibition study using conditionally immortalized rat BBB and inner BRB model cells, known as TR-BBB13 and TR-iBRB2 cells [48,49]. As shown in Table 1, ent-*cis*-**195A** exhibited an inhibitory effect on [^3^H]nicotine transport into TR-BBB13 cells and [^3^H]verapamil transport into TR-iBRB2 cells by more than 40%. In addition, the presence of *cis*-**211A** significantly attenuated [^3^H]nicotine and [^3^H]verapamil uptake by TR-BBB13 and TR-iBRB2 cells, respectively, by at least 29%. These results suggest that ent-*cis*-**195A** and *cis*-**211A** are recognized by the cationic drug transport systems at the BBB and inner BRB. It is possible that these derivatives reach the brain and retina via the cationic drug transport systems and show neuronal effects in the CNS.

In summary, we achieved the total syntheses of ent-*cis*-**195A** and *cis*-**211A** in a divergent process from the key and common intermediate **11**. The absolute stereochemistry of natural *cis*-**211A** was determined to be 2*R*, 4a*R*, 5*R*, 6*S*, and 8a*S* by comparison with the data obtained from our total synthesis. The inhibitory effects of ent-*cis*-**195A**, *cis*-**211A**, and 6-epi-**211A** on nicotinic ACh receptors were also investigated. The results showed that *cis*-**211A** and 6-epi-**211A** had better inhibitory effects on the α7-receptor than that of ent-*cis*-**195A**, and none of the compounds showed inhibitory effects on the α4β2-receptor at the same concentration. These results suggested that *cis*-**211A** and 6-epi-**211A** could be applied as important tools for studying the brain and nervous system. More interestingly, the absolute configuration of the decahydroquinoline nuclei of *cis*-**211A** was a mirror image of that of *cis*-**195A**, even though both alkaloids were isolated from *Oophaga* (*Dendrobates*) *pumilio* from Panama.

## 3. Materials and Methods

### 3.1. Chemistry

#### 3.1.1. General Information

Chemicals were purchased from Sigma–Aldrich, Merck (Darmstadt, Germany), FUJIFILM Wako Chemicals (Osaka, JAPAN), Nacalai Tesque, Tokyo Chemical Industry (Tokyo, Japan), and Kanto Chemical (Tokyo, Japan) and used without further purification. Column chromatography was done on Cica silica gel 60N (spherical, neutral; particle size, 63–210 nm, Kanto Chemical), while thin-layer chromatography was performed using Merck silica gel 60F_254_ plates. Melting points were taken on a Yanaco micromelting point apparatus and are uncorrected. The nuclear magnetic resonance (NMR) spectra were acquired in the specified solvent in JEOL JNM-A400 (400 and 100 MHz for ^1^H and ^13^C, respectively) or JEOL JNM-ECX500 (500 and 125 MHz for ^1^H and ^13^C, respectively). The chemical shifts (δ) are reported in ppm downfield from TMS, and coupling constants (*J*) are expressed in Hertz. IR spectra were measured with a JASCO FT/IR-460 Plus spectrophotometer (JASCO Corp., Tokyo, Japan). The low-resolution and high-resolution mass spectra were obtained with a Shimadzu GCMS-QP 500 mass spectrometer (Shimadzu Corp., Kyoto, Japan), JEOL D-200, or JEOL AX505 mass spectrometer (JEOL Ltd., Tokyo, Japan) in the electron impact mode at the ionization potential of 70 eV.

#### 3.1.2. Synthesis of (6*R*)-2-Phenylsulfanyl-6-propyl-piperidine-1,2-dicarboxylic Acid Dimethyl Ester (**3**)

To a stirred solution of **1** [39] (2.51 g, 10.39 mmol) in EtOAc (30 mL) was added 10% Pd/C (30 mg), and the resulting mixture was hydrogenated at 1 atm for 16 h. The catalyst was removed through a celite pad and washed with EtOAc (5 mL × 3). The filtrate and washings were combined and evaporated to give **2**, which was essentially pure and used directly in the next step. To a stirred solution of **2** in THF (30 mL) was added a solution of sodium bis(trimethylsilyl)amide (1.9 M in THF, 8.20 mL, 15.59 mmol) at −78 °C, and the reaction mixture was stirred at −78 °C for 30 min. To the reaction mixture was added a solution of diphenyl disulfide (3.40 g, 15.59 mmol) in THF (15 mL), and the resulting mixture was stirred at 0 °C for 30 min. The solvent was evaporated, and the residue was chromatographed on SiO_2_ (50 g, acetone/*n*-hexane = 1/30) to give **3** (3.39 g, 9.66 mmol, 93% in 2 steps) as a yellow oil as a mixture of diastereomers.

^1^H-NMR (400 MHz CDCl_3_) δ: 0.90 and 0.94 (3H, each t, *J* = 7.2 Hz), 1.28–1.79 (8H, m), 1.90–1.98 (1H, m), 2.26–2.38 (1H, m), 3.49 and 3.62 (3H, each s), 3.73 and 3.74 (3H, each s), 4.06–4.20 (1H, m), 7.29–7.35 (3H, m), 7.73–7.78 (2H, m).

#### 3.1.3. Synthesis of (6*R*)-6-Propyl-5,6-dihydro-4*H*-pyridine-1,2-dicarboxylic Acid Dimethyl Ester (**4**)

To a stirred solution of **3** (1.27 g, 3.61 mmol) in CH_2_Cl_2_ (12 mL) was added 2,6-lutidine (0.84 mL, 9.03 mmol), and then *m*CPBA (70%, 1.50 g, 8.67 mmol) was added to the reaction mixture in four portions in 15 min intervals at 0 °C. The resulting mixture was stirred at room temperature for 8 h. The reaction was quenched with 10% Na_2_S_2_O_3_ in sat. NaHCO_3_ (aq.) (25 mL), and the aqueous mixture was diluted with EtOAc. The layers were separated, and the aqueous layer was extracted with EtOAc (5 mL × 3). The organic layer and extracts were combined and washed with brine, 10% HCl (aq.), and brine, successively. The organic layer was dried and evaporated to give a pale yellow oil, which was chromatographed on SiO_2_ (20 g, acetone/*n*-hexane = 1/30) to give **4** (871 mg, 3.61 mmol, 100%) as pale yellow oil.

^1^H-NMR (400 MHz CDCl_3_) δ: 0.93 (3H, t, *J* = 7.3 Hz), 1.17–1.28 (1H, m), 1.37-1.58 (3H, m), 1.69–1.76 (1H, m), 1.79–1.89 (1H, m), 2.15–2.22 (2H, m), 3.70 (3H, s), 3.76 (3H, s), 4.42 (1H, br), 6.06 (1H, t, *J* = 3.6 Hz); ^13^C-NMR (125 MHz CDCl_3_) δ: 13.75, 19.10, 19.53, 25.70, 31.62, 50.99, 51.87, 52.85, 122.08, 129.87, 154.63, 165.56; IR (neat): 1231, 1275, 1330, 1442, 1714, 1733 cm^−1^; MS (EI): *m*/*z* 241 (M^+^); HRMS (EI) Calcd for C_12_H_19_NO_4_ 241.1314 (M^+^); Found 241.1315; [α]_D_^19^ − 68.0 (*c* 1.00, CHCl_3_).

#### 3.1.4. Synthesis of (2*R*, 3*S*, 6*R*)-6-Propyl-3-vinyl-piperidine-1,2-dicarboxylic Acid Dimethyl ester (**5**)

To a stirred solution of CuI (1.31 g, 6.90 mmol) in Et_2_O (15 mL) was added a solution of vinyl lithium, prepared from tetravinyltin (0.61 mL, 3.45 mmol) and MeLi (1.13 M in Et_2_O, 12.20 mL, 13.80 mmol) in Et_2_O (15 mL) at 0 °C for 30 min, at −78 °C, and the reaction mixture was warmed to -35 °C for 30 min. The reaction mixture was recooled to −78 °C, and a solution of **4** (555 mg, 2.30 mmol) in Et_2_O (7 mL) was added to the reaction mixture. The resulting mixture was gradually warmed to 0 °C and stirred at the same temperature for 1 h. The reaction was quenched with sat. NH_4_Cl (aq.) (30 mL). The aqueous mixture was diluted with CH_2_Cl_2_ (30 mL), and the resulting mixture was filtered. The filtrate was separated, and the aqueous layer was extracted with CH_2_Cl_2_ (10 mL × 3). The organic layer and extracts were combined, dried, and evaporated to give a colorless oil, which was chromatographed on SiO_2_ (20 g, acetone/*n*-hexane = 1/30) to give **5** (613 mg, 2.28 mmol, 99%) as a colorless oil.

^1^H-NMR (400 MHz CDCl_3_) δ: 0.90 (3H, t, *J* = 7.0 Hz), 1.25–1.56 (6H, m), 1.78–1.92 (2H, m), 3.08 (1H, br), 3.71 (3H, s), 3.74 (3H, s), 4.17–4.18 (1H, m), 4.88 (1H, br), 5.09–5.15 (2H, m), 5.81 (1H, ddd, *J* = 17.1, 10.7, 6.4 Hz); ^13^C-NMR (125 MHz CDCl_3_) δ: 13.86, 19.88, 21.03, 22.45, 34.59, 36.52, 50.96, 51.99, 52.75, 55.07, 115.16, 139.00, 157.08, 172.86; IR (neat): 1200, 1340, 1363, 1448, 1506, 1558, 1683, 1699, 1734 cm^−1^; MS (EI): *m*/*z* 269 (M^+^); HRMS (EI) Calcd for C_14_H_23_NO_4_ 269.1627 (M^+^); Found 269.1631; [α]_D_^25^ + 53.6 (*c* 1.00, CHCl_3_).

#### 3.1.5. Synthesis of (2*S*, 3*S*, 6*R*)-2-Methoxycarbonylmethyl-6-propyl-3-vinyl-piperidine-1-carboxylic Acid Methyl Ester (**6**)

To a stirred solution of **5** (428 mg, 1.59 mmol) in MeOH (6 mL) and H_2_O (2 mL) was added LiOH·H_2_O (266 mg, 6.36 mmol), and the resulting mixture was refluxed for 2 h. After cooling, MeOH was evaporated, and the residue was acidified with 10% HCl (aq.) (5 mL). The aqueous mixture was extracted with EtOAc (3 mL × 5). The organic extracts were combined, dried, and evaporated to give a yellow oil, which was used directly in the next step. To a stirred solution of the above oil in THF (10 mL) were added ClCO_2_Et (0.18 mL, 1.91 mmol) and Et_3_N (0.27 mL, 1.91 mmol) at 0 °C, and the resulting mixture was stirred at 0 °C for 1 h. The reaction mixture was diluted with Et_2_O (3 mL), and Et_3_N·HCl was filtered off. The filtrate was evaporated to give a yellow oil, which was used directly in the next step. To a stirred solution of the above oil in Et_2_O (10 mL) was added a solution of CH_2_N_2_ in Et_2_O at 0 °C, and the reaction mixture was stirred at room temperature for 16 h. The solvent was evaporated to give a yellow oil, which was dissolved in MeOH (10 mL). To the MeOH solution were added AgCO_2_Ph (37 mg, 0.16 mmol) and Et_3_N (0.45 mL, 3.18 mmol), and the resulting mixture was stirred at room temperature for 24 h. The reaction mixture was diluted with Et_2_O, and the insoluble material was filtered off. The filtrate was evaporated to give a black oil, which was chromatographed on SiO_2_ (20 g, EtOAc/*n*-hexane = 1/30) to give **6** (409 mg, 1.45 mmol, 91% in 4 steps) as a colorless oil.

^1^H-NMR (400 MHz CDCl_3_) δ: 0.92 (3H, t, *J* = 7.3 Hz), 1.20-1.42 (4H, m), 1.43-1.52 (2H, m), 1.78-1.92 (2H, m), 2.32 (1H, br), 2.54 (1H, dd, *J* = 14.9, 4.8 Hz), 2.65 (1H, dd, *J* = 14.9, 10.1 Hz), 3.66 (3H, s), 3.68 (3H, s), 4.12 (1H, br), 4.61 (1H, br), 5.06 (1H, dt, *J* = 10.6, 1.4 Hz), 5.09 (1H, dt, *J* = 17.2, 1.4 Hz), 5.84 (1H, ddd, *J* = 17.2, 10.6, 6.6 Hz); ^13^C-NMR (125 MHz CDCl_3_) δ: 13.96, 20.05, 20.32, 22.18, 37.46, 39.73, 39.90, 50.73, 50.92, 51.67, 52.62, 115.07, 140.04, 156.76, 171.64; IR (neat): 1101, 1363, 1443, 1696, 1740 cm^−1^; MS (EI): *m*/*z* 283 (M^+^); HRMS (EI) Calcd for C_15_H_25_NO_4_ 283.1784 (M^+^); Found 269.1780; [α]_D_^19^ -31.4 (*c* 1.00, CHCl_3_).

#### 3.1.6. Synthesis of (2*S*, 3*S*, 6*R*)-2-[(Methoxy-methyl-carbamoyl)-methyl]-6-propyl-3-vinyl-piperidine-1-carboxylic Acid Methyl Ester (**7**)

To a stirred solution of **6** (571 mg, 2.02 mmol) in MeOH (4.5 mL) and H_2_O (1.5 mL) was added LiOH·H_2_O (338 mg, 8.06 mmol), and the resulting mixture was refluxed for 2 h. After cooling, MeOH was evaporated, and the residue was acidified with 10% HCl (aq.) (3 mL). The aqueous mixture was extracted with EtOAc (3mL × 5). The organic extracts were combined, dried, and evaporated to give a yellow oil, which was used directly in the next step. To a stirred solution of the above oil in CH_2_Cl_2_ (7 mL) was added 1,1-carbonyldiimidazole (457 mg, 2.82 mmol) at 0 °C, and the reaction mixture was stirred for 30 min. To the reaction mixture were added MeO(Me)NH·HCl (275 mg, 2.82 mmol) and Et_3_N (0.40 mL, 2.82 mmol) at 0 °C, and the resulting mixture was stirred at room temperature for 16 h. The solvent was evaporated, and the residue was chromatographed on SiO_2_ (10 g, acetone/*n*-hexane = 1/7) to give **7** (610 mg, 1.95 mmol, 97% in 2 steps) as a colorless oil.

^1^H-NMR (500 MHz CDCl_3_) δ: 0.91 (3H, t, *J* = 7.3 Hz), 1.16–1.43 (4H, m), 1.48 (2H, q, *J* = 6.0 Hz), 1.76–1.92 (2H, m), 2.37 (1H, br), 2.53–2.56 (1H, m), 2.80 (1H, m), 3.12 (3H, br), 3.65 (3H, s), 3.67 (3H, s), 4.12 (1H, br), 4.63 (1H, br), 5.04 (1H, dd, *J* = 10.7, 1.4 Hz), 5.07 (1H, dd, *J* = 17.2, 1.4 Hz), 5.84 (1H, ddd, *J* = 17.2, 10.7, 1.4 Hz); ^13^C-NMR (125 MHz CDCl_3_) δ: 13.96, 19.85, 20.26, 22.15, 29.20, 32.12, 37.36, 39.15, 50.20, 50.55, 52.51, 61.22, 114.82, 140.30, 156.75, 172.01; IR (neat): 1100, 1348, 1362, 1444, 1667, 1694, 1698 cm^−1^; MS (EI): *m*/*z* 312 (M^+^); HRMS (EI) Calcd for C_16_H_28_N_2_O_4_ 312.2049 (M^+^); Found 312.2046; [α]_D_^23^ − 36.0 (*c* 1.00, CHCl_3_).

#### 3.1.7. Synthesis of (2*S*, 3*S*, 6*R*)-2-(2-Oxo-propyl)-6-propyl-3-vinyl-piperidine-1-carboxylic Acid Methyl Ester (**8**)

To a stirred solution of **7** (188 mg, 0.60 mmol) in THF (3 mL) was added a solution MeMgBr (0.91 M in THF, 0.97 mL, 0.72 mmol) at 0 °C, and the resulting mixture was stirred at 0 °C for 1 h. The reaction was quenched with sat. NH_4_Cl (aq.) (5 mL). The layers were separated, and the aqueous layer was extracted with CH_2_Cl_2_ (5 mL × 3). The organic layer and extracts were combined, dried, and evaporated to give a colorless oil, which was chromatographed on SiO_2_ (7 g, acetone/*n*-hexane = 1/7) to give **8** (160 mg, 0.60 mmol, 99%) as a colorless oil.

^1^H-NMR (500 MHz CDCl_3_) δ: 0.93 (3H, t, *J* = 7.3 Hz), 1.19–1.62 (6H, m), 1.79–1.91 (2H, m), 2.18 (3H, s), 2.24 (1H, br), 2.59–2.63 (1H, dd, *J* = 12.0, 2.8 Hz), 2.70-2.79 (1H, dd, *J* = 12.0, 8.4 Hz), 3.69 (3H, s), 4.12 (1H, br), 4.62–4.64 (1H, m), 5.07 (1H, dd, *J* = 10.6, 1.5 Hz), 5.09 (1H, dd, *J* = 17.2, 1.5 Hz), 5.86 (1H, ddd, *J* = 17.2, 10.6, 1.5 Hz); ^13^C-NMR (125 MHz CDCl_3_) δ: 13.97, 19.82, 20.29, 22.00, 29.86, 37.30, 39.59, 49.44, 50.00, 50.26, 52.56, 115.08, 140.07, 156.74, 206.60; IR (neat): 1102, 1277, 1361, 1407, 1443, 1640, 1694 cm^−1^; MS (EI): *m*/*z* 267 (M^+^); HRMS (EI) Calcd for C_15_H_25_NO_3_ 267.1834 (M^+^); Found 267.1835; [α]_D_^19^ − 70.0 (*c* 1.00, CHCl_3_).

#### 3.1.8. Synthesis of (2*S*, 3*S*, 6*R*)-3-Formyl-2-(2-oxo-propyl)-6-propyl-piperidine-1-carboxylic Acid Methyl Ester (**9**)

To a stirred solution of **8** (368 mg, 1.38 mmol) in 1,4-dioxane (6 mL) and H_2_O (2 mL) was added 2,6-lutidine (0.32 mL, 2.75 mmol), OsO_4_ (2% aqueous solution, 1.7 mL, 0.14 mmol) and NaIO_4_ (1.18 g, 5.51 mmol) at 0 °C, and the resulting mixture was stirred at room temperature for 3 h. The reaction was quenched with 10% Na_2_S_2_O_3_ in sat. NaHCO_3_ (aq.) (10 mL), and the aqueous mixture was diluted with CH_2_Cl_2_. The layers were separated, and the aqueous layer was extracted with CH_2_Cl_2_ (5 mL × 3). The organic layer and extracts were combined; washed with brine, 10% HCl (aq.), and brine, successively; dried; and evaporated to give a yellow oil, which was chromatographed on SiO_2_ (10 g, acetone/*n*-hexane = 1/10) to give **9** (349 mg, 1.29 mmol, 94%) as a colorless oil.

^1^H-NMR (500 MHz CDCl_3_) δ: 0.90 (3H, t, *J* = 7.3 Hz), 1.16–1.26 (1H, m), 1.26–1.36 (1H, m), 1.38–1.57 (4H, m), 1.74–1.82 (1H, m), 1.93–1.99 (1H, m), 2.17 (3H, s), 2.37 (1H, br), 2.70–2.80 (2H, m), 3.67 (3H, s), 4.06 (1H, br), 5.15 (1H, br), 9.66 (1H, s); ^13^C-NMR (125 MHz CDCl_3_) δ: 13.94, 14.37, 20.22, 23.39, 30.15, 36.61, 45.10, 48.19, 48.90, 49.93, 52.74, 156.29, 202.79, 206.29; IR (neat): 1100, 1328, 1354, 1447, 1684, 1694, 1717, 2873, 2957 cm^−1^; MS (EI): *m*/*z* 269 (M^+^); HRMS (EI) Calcd for C_14_H_23_NO_4_ 269.1627 (M^+^); Found 269.1629; [α]_D_^23^ −114.8 (*c* 1.00, CHCl_3_).

#### 3.1.9. Synthesis of (2*R*, 4a*R*, 8a*S*)-7-Oxo-2-propyl-3,4,4a,7,8,8a-hexahydro-2*H*-quinoline-1-carboxylic Acid Methyl Ester (**10c**)

To a stirred solution of **9** (349 mg, 1.30 mmol) in benzene (30 mL) was added DBU (0.78 mL, 5.18 mmol) and MS 4 Å (50 mg), and the resulting mixture was refluxed for 48 h. After cooling, benzene was evaporated, and the residue was acidified with 10% HCl (aq.) (5 mL). The aqueous mixture was extracted with EtOAc (3mL × 5). The organic extracts were combined, dried, and evaporated to give a brown oil, which was chromatographed on SiO_2_ (25 g, EtOAc/*n*-hexane = 1/10) to give **10c** (235 mg, 0.94 mmol, 72%) as a yellow oil.

^1^H-NMR (500 MHz CDCl_3_) δ: 0.91 (3H, t, *J* = 7.2 Hz), 1.24-1.41 (2H, m), 1.44–1.55 (2H, m), 1.59 (1H, td, *J* = 10.0, 2.4 Hz), 1.67 (1H, tdd, *J* = 10.0, 4.8, 2.4 Hz), 1.73–1.78 (1H, m), 1.78–1.83 (1H, m), 2.40–2.45 (1H, m), 2.61 (2H, br), 3.70 (3H, s), 4.25 (1H, br), 4.63 (1H, br), 6.13 (1H, d, *J* = 9.7 Hz), 6.77 (1H, dd, *J* = 9.7, 5.7 Hz); ^13^C-NMR (125 MHz CDCl_3_) δ: 13.88, 19.95, 20.24, 27.09, 36.53, 37.00, 40.30, 48.43, 49.73, 52.60, 128.64, 152.18, 156.04, 198.22; IR (neat): 771, 1089, 1115, 1246, 1275, 1314, 1444, 1685, 2934 cm^−1^; MS (EI): *m*/*z* 251 (M^+^); HRMS (EI) Calcd for C_14_H_21_NO_3_ 251.1521 (M^+^); Found 251.1522; [α]_D_^24^ + 29.9 (*c* 1.00, CHCl_3_).

#### 3.1.10. Synthesis of (2*R*, 4a*R*, 5*R*, 8a*R*)-5-Methyl-2-propyl-7-trifluoromethane-sulfonyloxy-3,4,4a,5,8,8a-hexahydro-2*H*-quinoline-1-carboxylic Acid Methyl Ester (**11**)

To a stirred solution of CuI (147 mg, 0.77 mmol) in Et_2_O (3 mL) was added a solution of MeLi (1.17 M in Et_2_O, 1.32 mL, 1.54 mmol) at −78 °C, and the reaction mixture was warmed to 0 °C for 30 min. The reaction mixture was recooled to −78 °C, and a solution of **10c** (97 mg, 0.39 mmol) in Et_2_O (3 mL) was added to the reaction mixture. The reaction mixture was gradually warmed to 0 °C, and then a solution of Comins’ reagent (303 mg, 0.77 mmol) in Et_2_O (3 mL) was added to the reaction mixture. The resulting mixture was stirred at room temperature for 2 h, and the reaction was quenched with sat. NH_4_Cl (aq.) (5 mL). The aqueous mixture was diluted with CH_2_Cl_2_ (5 mL), and the resulting suspension was filtered. The filtrate was separated, and the aqueous layer was extracted with CH_2_Cl_2_ (3 mL × 3). The filtrate and extracts were combined, dried, and evaporated to give a colorless oil, which was chromatographed on SiO_2_ (10 g, acetone/*n*-hexane = 1/50) to give **11** (144 mg, 0.36 mmol, 94%) as a colorless oil.

^1^H-NMR (400 MHz CDCl_3_) δ: 0.90 (3H, t, *J* = 7.2 Hz), 1.12 (3H, d, *J* = 7.2 Hz), 1.19–1.39 (4H, m), 1.39–1.50 (1H, m), 1.50–1.72 (4H, m), 2.26 (1H, t, *J* = 6.0 Hz), 2.50 (2H, br), 3.69 (3H, s), 4.10 (1H, br), 4.50 (1H, br), 5.66 (1H, d, *J* = 6.0 Hz); ^13^C-NMR (100 MHz CDCl_3_) δ: 13.83, 19.15, 20.68, 21.49, 27.88, 29.67, 35.23, 38.04, 40.26, 46.91, 50.41, 52.63, 118.45 (q, *J* = 318.5 Hz), 121.19, 144.70, 156.65; IR (neat): 1144, 1209, 1246, 1419, 1445, 1699, 2936, 2959 cm^−1^; MS (EI): *m*/*z* 399 (M^+^); HRMS (EI) Calcd for C_16_H_24_F_3_NO_5_S 399.1327 (M^+^); Found 399.1337; [α]_D_^23^ + 58.6 (*c* 1.35, CHCl_3_).

#### 3.1.11. Synthesis of (2*R*, 4a*R*, 5*R*, 8a*R*)-5-Methyl-2-propyl-octahydro-quinoline-1-carboxylic Acid Methyl Ester (**12**)

To a stirred solution of **11** (60 mg, 0.15 mmol) in MeOH (3 mL) was added 20% Pd(OH)_2_/C (5 mg), and the resulting mixture was hydrogenated at 1 atm for 16 h. The catalyst was removed through a celite pad and washed with MeOH (3 mL × 3). The filtrate and washings were combined and evaporated to give a pale yellow oil, which was chromatographed on SiO_2_ (8 g, acetone/*n*-hexane = 1/30) to give **12** (33 mg, 0.13 mmol, 88%) as a colorless oil.

^1^H-NMR (500 MHz CDCl_3_) δ: 0.90 (3H, t, *J* = 7.2 Hz), 1.06 (3H, d, *J* = 6.9 Hz), 1.17–1.68 (14H, m), 1.79–1.88 (2H, m), 3.70 (3H, s), 4.06 (1H, br), 4.22 (1H, br); ^13^C-NMR (125 MHz CDCl_3_) δ: 14.17, 19.41, 20.41, 20.72, 21.36, 26.82, 28.19, 28.57, 34.61, 37.81, 42.19, 49.81, 50.49, 52.36, 156.65; IR (neat): 1303, 1317, 1443, 1695, 2864, 2929, 2955 cm^−1^; MS (EI) *m*/*z* 253 (M^+^); HRMS (EI) Calcd for C_15_H_27_NO_2_ 253.2042 (M^+^); Found 253.2043; [α]_D_^17^ −20.2 (*c* 1.00, CHCl_3_).

#### 3.1.12. Synthesis of (2*R*, 4a*R*, 5*R*, 8a*R*)-5-Methyl-2-propyldecahydroquinoline (ent-*cis*-**195A**)

To a stirred solution of **12** (42 mg, 0.17 mmol) in CHCl_3_ (3 mL) was added NaI (197 mg, 1.32 mmol) and TMSCl (0.10 mL, 0.83 mmol), and the resulting mixture was heated to 50 °C for 24 h. After cooling, the reaction was quenched with 10% Na_2_S_2_O_3_ in sat. NaHCO_3_ (aq.) (3 mL), and aqueous mixture was extracted with CH_2_Cl_2_ (2 mL x 10). The organic extracts were combined, dried, and evaporated to give a pale yellow oil, which was chromatographed on SiO_2_ (3 g, MeOH/CH_2_Cl_2_ = 1/20) to give ent-*cis*-**195A** (30 mg, 0.15 mmol, 88%) as a pale yellow oil. ent-*cis*-**195A^.^**HCl was obtained in quantitative yield by treatment with HCl (ca. 1 mol/L in Et_2_O) followed by evaporation.

ent-*cis*-**195A**: ^1^H-NMR (400 MHz CDCl_3_) δ: 0.83 (3H, d, *J* = 6.6 Hz), 0.90 (3H, t, *J* = 7.0 Hz), 0.95–1.02 (1H, m), 1.03–1.14 (2H, m), 1.25–1.49 (8H, m), 1.52–1.70 (4H, m), 1.79–1.90 (1H, m), 1.90–1.97 (1H, m), 2.53 (1H, dtd, *J* = 11.4, 5.8, 2.8 Hz), 2.84 (1H, q, *J* = 2.8 Hz); ^13^C-NMR (100 MHz CDCl_3_) δ: 14.30, 19.14, 19.91, 21.21, 26.99, 27.24, 27.35, 33.28, 35.88, 39.59, 42.49, 55.96, 57.72; IR (neat): 857, 890, 962, 1024, 1044, 1081, 1124, 1167, 1256, 1317, 1348, 1380, 1451, 1734, 2803, 2873, 2935 cm^−1^; MS (EI) *m*/*z* 195 (M^+^); HRMS (EI) Calcd for C_13_H_25_N 195.1987 (M^+^); Found 195.1985; ent-*cis*-**195A^.^**HCl: [α]_D_^20^ + 12.7 (*c* 0.35, MeOH).

The ^1^H- and ^13^C-NMR spectra and optical rotation of the synthetic sample were identical with those of the literature data.

^1^H-NMR (500 MHz CDCl_3_) δ: 0.83 (3H, d, *J* = 6.6 Hz), 0.90 (3 H, t, *J* = 7.0 Hz), 0.94–1.03 (1H, m), 1.05–1.14 (2H, m), 1.22–1.49 (9H, m), 1.50–1.71 (4H, m), 1.77–1.90 (1H, m), 1.90–1.99 (1H, m), 2.53 (1H, dtd, J = 11.4, 5.8, 2.7 Hz), 2.84 (1H, q, *J* = 2.8 Hz); ^13^C-NMR (75 MHz CDCl_3_) δ: 14.5 (CH_3_), 19.3 (CH_2_), 20.1 (CH_3_), 21.4 (CH_2_), 27.2 (CH_2_), 27.54 (CH), 27.55 (CH_2_), 33.6 (CH_2_), 36.1 (CH_2_), 39.9 (CH_2_), 42.8 (CH), 56.1 (CH), 57.9 (CH); ent-*cis*-**195A^.^**HCl: [α]_D_^20^ +12.9 (*c* 0.36, MeOH) [22].

#### 3.1.13. Synthesis of (2*R*, 4a*R*, 5*R*, 8a*R*)-5-Methyl-2-propyl-3,4,4a,5,8,8a-hexahydro-2H-quinoline-1-carboxylic Acid Methyl Ester (**13**)

To a stirred solution of **11** (144 mg, 0.36 mmol) in THF (3 mL) was added PPh_3_ (7 mg, 0.03 mmol) and Pd(OAc)_2_ (3 mg, 0.01 mmol) at room temperature, and the reaction mixture was heated to 25 °C for 10 min. A solution of Et_3_N (0.11 mL, 0.81 mmol) and formic acid (0.03 mL, 0.80 mmol) in THF (1 mL) was added to the reaction mixture, and the resulting mixture was refluxed for 18 h. The reaction was quenched with brine (5 mL). The layers were separated, and the aqueous layer was extracted with CH_2_Cl_2_ (3 mL × 3). The organic layer and extracts were combined, dried, and evaporated to give a colorless oil, which was chromatographed on SiO_2_ (8 g, acetone/*n*-hexane = 1/50) to give **13** (85 mg, 0.36 mmol, 100%) as a colorless oil.

^1^H-NMR (400 MHz CDCl_3_) δ: 0.89 (3H, t, *J* = 7.2 Hz), 1.05 (3H, d, *J* = 6.4 Hz), 1.23–1.66 (9H, m), 2.03 (1H, m), 2.15 (2H, m), 3.67 (3H, s), 4.07 (1H, br), 4.35 (1H, br), 5.44–5.53 (2H, m); ^13^C-NMR (100 MHz CDCl_3_) δ: 14.02, 20.77, 21.96, 22.42, 27.34, 28.21, 29.67, 37.03, 41.33, 47.11, 50.53, 52.33, 122.82, 130.53, 156.39; IR (neat): 1093, 1304, 1320, 1444, 1695, 2871, 2930, 2955 cm^−1^; MS (EI) *m*/*z* 251 (M^+^); HRMS (EI) Calcd for C_15_H_25_NO_2_ 251.1885 (M^+^); Found 251.1877; [α]_D_^23^ +57.4 (*c* 0.90, CHCl_3_).

#### 3.1.14. Synthesis of (2*R*, 4a*R*, 5*R*, 6*R*, 8a*R*)-6-Hydroxy-5-methyl-2-propyl-octahydro-quinoline-1-carboxylic Acid Methyl Ester (**15**)

To a stirred solution of **13** (18 mg, 0.08 mmol) in toluene (1.5 mL) was added BH_3_·SMe_2_ (0.02 mL, 0.23 mmol) at 0 °C. The reaction mixture was stirred at room temperature for 24 h and then cautiously quenched with 10% NaOH (0.25 mL) at 0 °C, followed by the slow addition of H_2_O_2_ (30%, 0.25 mL) at 0 °C. The reaction mixture was stirred at room temperature for 24 h. The layers were separated, and the aqueous layer was extracted with CH_2_Cl_2_ (3 mL × 3). The organic layer and extracts were combined, dried, and evaporated to give a colorless oil, which was chromatographed on SiO_2_ (5 g, acetone/*n*-hexane = 1/10) to give **15** (10 mg, 0.04 mmol, 50%) as a colorless oil and the inseparable mixture of alcohols **16** and **17** (9 mg, 0.03 mmol, 45%) as a colorless oil.

**15**: ^1^H-NMR (400 MHz CDCl_3_) δ: 0.91 (3H, t, *J* = 7.6 Hz), 1.05 (3H, d, *J* = 7.6 Hz), 1.20–1.26 (1H, m), 1.28–1.36 (2H, m), 1.46–1.68 (7H, m), 1.78 (1H, qdd, *J* = 14.3, 3.9, 2.8 Hz), 1.88 (1H, qd, *J* = 7.6, 1.7 Hz), 2.01 (1H, qd, *J* = 13.2, 3.9 Hz), 2.28 (1H, qd, *J* = 13.6, 3.2 Hz), 3.67 (3H, s), 4.02–4.10 (1H, m), 4.18–4.25 (1H, m); ^13^C-NMR (100 MHz CDCl_3_) δ: 14.05, 18.89, 20.56, 22.77, 28.35, 28.64, 29.68, 37.33, 41.32, 41.87, 49.86, 50.51, 52.32, 70.97, 156.69; IR (neat): 1319, 1447, 1456, 1670, 1697, 2872, 2934, 2957 cm^−1^; MS (EI) *m*/*z* 269 (M^+^); HRMS (EI) Calcd for C_15_H_27_NO_3_ 269.1991 (M^+^); Found 269.1990; [α]_D_^25^ −17.1 (*c* 0.80, CHCl_3_).

#### 3.1.15. Synthesis of (2*R*, 4a*R*, 5*R*, 8a*R*)-5-Methyl-6-oxo-2-propyl-octahydro-quinoline-1-carboxylic Acid Methyl Ester (**19**)

To a stirred solution of **15** (46 mg, 0.17 mmol) in CH_2_Cl_2_ (1.5 mL) and sat. NaHCO_3_ (aq.) (1.5 mL) was added NaOCl·5H_2_O (55 mg, 0.34 mmol) and AZADOL^®^ (0.3 mg, 0.0017 mmol) at 0 °C, and the resulting mixture was stirred at room temperature for 1 h. The layers were separated, and the aqueous layer was extracted with CH_2_Cl_2_ (1 mL x 3). The organic layer and extracts were combined, dried, and evaporated to give a yellow oil, which was chromatographed on SiO_2_ (8 g, acetone/*n*-hexane = 1/5) to give **19** (43 mg, 0.16 mmol, 98%) as pale yellow oil.

^1^H-NMR (400 MHz CDCl_3_) δ: 0.88 (3H, t, *J* = 6.8 Hz), 1.24 (3H, d, *J* = 7.6 Hz), 1.26–1.69 (8H, m), 1.76–1.84 (1H, m), 1.92–2.00 (1H, m), 2.06 (1H, qd, *J* = 12.8, 4.8 Hz), 2.16–2.22 (1H, m), 2.26–2.33 (1H, m), 2.60 (1H, td, *J* = 14.8, 6.8 Hz), 3.69 (3H, s), 4.05–4.12 (1H, m), 4.58–4.61 (1H, m); ^13^C-NMR (100 MHz CDCl_3_) δ: 13.99, 17.20, 20.53, 21.91, 27.40, 27.53, 29.68, 36.94, 37.45, 43.75, 48.89, 50.12, 50.45, 52.58, 156.57, 213.93; IR (neat): 1093, 1133, 1189, 1244, 1275, 1312, 1348, 1409, 1444, 1699, 1717, 2855, 2929, 2954 cm^−1^; MS (EI) *m*/*z* 267 (M^+^); HRMS (EI) Calcd for C_15_H_25_NO_3_ 267.1834 (M^+^); Found 267.1834; [α]_D_^25^ −21.4 (*c* 0.50, CHCl_3_).

#### 3.1.16. Synthesis of (2*R*, 4a*R*, 5*R*, 6*S*, 8a*R*)-6-Hydroxy-5-methyl-2-propyl-octahydro-quinoline-1-carboxylic Acid Methyl Ester (**16**)

To a stirred solution of **19** (18 mg, 0.07 mmol) in THF (2 mL) was added a solution of Super-Hydride^®^ (1.0 M in THF, 0.20 mL, 0.20 mmol) at 0 °C, and the resulting mixture was stirred at room temperature for 1 h. The reaction was quenched with sat. NH_4_Cl (aq.) (2 mL). The layers were separated, and the aqueous layer was extracted with CH_2_Cl_2_ (1 mL × 3). The organic layer and extracts were combined, dried, and evaporated to give a colorless oil, which was chromatographed on SiO_2_ (5 g, acetone/*n*-hexane = 1/5) to give **16** (16 mg, 0.06 mmol, 89%) as a colorless oil and **15** (2 mg, 0.01 mmol, 11%) as a colorless oil.

^1^H-NMR (400 MHz CDCl_3_) δ: 0.91 (3H, t, *J* = 7.2 Hz), 1.06 (3H, d, *J* = 7.2 Hz), 1.23–1.71 (13H, m), 1.83 (1H, qd, *J* = 13.6, 2.8 Hz), 1.99 (1H, t, *J* = 6.0 Hz), 3.68 (3H, s), 4.02–4.11 (1H, m), 4.19–4.29 (1H, m); ^13^C-NMR (100 MHz CDCl_3_) δ: 12.22, 14.03, 20.58, 21.03, 28.12, 28.64, 29.68, 37.58, 40.53, 43.06, 48.75, 50.33, 52.38, 68.66, 156.63; IR (neat): 1097, 1319, 1448, 1670, 2870, 2932, 2955 cm^−1^; MS (EI) *m*/*z* 269 (M^+^); HRMS (EI) Calcd for C_15_H_27_NO_3_ 269.1991 (M^+^); Found 269.1990; [α]_D_^25^ −16.5 (*c* 0.50, CHCl_3_).

#### 3.1.17. Synthesis of (2*R*, 4a*R*, 5*R*, 6*S*, 8a*R*)-5-Methyl-2-propyldecahydroquinoline-6-ol (*cis*-**211A**)

To a stirred solution of **16** (15 mg, 0.06 mmol) in MeCN (2 mL) was added NaI (67 mg, 0.45 mmol) and TMSCl (0.04 mL, 0.28 mmol), and the resulting mixture was refluxed for 1 h. After cooling, the reaction was quenched with 10% Na_2_S_2_O_3_ in sat. NaHCO_3_ (aq.) (2 mL). The layers were separated, and the aqueous layer was extracted with CH_2_Cl_2_ (1 mL x 10). The organic layer and extracts were combined, dried, and evaporated to give a pale yellow oil, which was chromatographed on SiO_2_ (3 g, MeOH/CH_2_Cl_2_ = 1/5) to give *cis*-**211A** (13 mg, 0.06 mmol, 100%) as pale yellow oil.

^1^H-NMR (400 MHz CDCl_3_) δ: 0.90 (3H, t, *J* = 6.4 Hz), 0.95 (3H, d, *J* = 6.8 Hz), 1.10–1.18 (2H, m), 1.27–1.45 (6H, m), 1.54–1.66 (3H, m), 1.80–1.87 (1H, m), 1.91–2.03 (2H, m), 2.06–2.15 (1H, m), 2.53–2.62 (1H, m), 2.89 (1H, br), 3.83 (1H, br); ^13^C-NMR (100 MHz CDCl_3_) δ: 14.22, 15.82, 19.09, 26.37, 26.45, 28.08, 29.68, 31.02, 34.55, 39.09, 55.94, 57.95, 72.11; IR (neat): 753, 812, 883, 946, 997, 1029, 1100, 1158, 1191, 1257, 1317, 1339, 1376, 1444, 2806, 2879, 2934, 3659 cm^−1^; MS (EI) *m*/*z* 211 (M^+^); HRMS (EI) Calcd for C_13_H_25_NO 211.1936 (M^+^); Found 211.1943; [α]_D_^25^ −11.5 (*c* 1.00, CHCl_3_).

The ^1^H- and ^13^C-NMR spectra and optical rotation of the synthetic sample were identical with those of the literature data.

^1^H-NMR (400 MHz CDCl_3_) δ: 0.90 (3H, t), 0.95 (3H, d), 1.06–1.16 (2H, m), 1.29–1.43 (6H, m), 1.56–1.67 (3H, m), 1.81–1.87 (1H, m), 1.91–2.03 (2H, m), 2.06–2.15 (1H, m), 2.53–2.61 (1H, m), 2.88 (1H, br), 3.82 (1H, br); ^13^C-NMR (100 MHz CDCl_3_) δ: 14.3, 15.8, 19.1, 26.7, 26.8, 26.9, 28.9, 31.1, 34.9, 39.3, 55.8, 57.8, 72.3; [α]_D_^25^ −11.7 (*c* 1.00, CHCl_3_) [23].

#### 3.1.18. Synthesis of (2*R*, 4a*R*, 5*R*, 6*R*, 8a*R*)-5-Methyl-2-propyldecahydroquinoline-6-ol (6-epi-**211A**)

To a stirred solution of **15** (18 mg, 0.07 mmol) in MeCN (2 mL) was added NaI (80 mg, 0.54 mmol) and TMSCl (0.04 mL, 0.33 mmol), and the resulting mixture was refluxed for 1 h. After cooling, the reaction was quenched with 10% Na_2_S_2_O_3_ in sat. NaHCO_3_ (aq.) (2 mL). The layers were separated, and the aqueous layer was extracted with CH_2_Cl_2_ (1 mL × 10). The organic layer and extracts were combined, dried, and evaporated to give a pale yellow oil, which was chromatographed on SiO_2_ (3 g, MeOH/CH_2_Cl_2_ = 1/5) to give 6-epi-**211A** (10 mg, 0.05 mmol, 72%) as pale yellow oil.

^1^H-NMR (400 MHz CDCl_3_) δ: 0.95 (3H, t, *J* = 7.2 Hz), 1.03–1.05 (1H, m), 1.07 (3H, d, *J* = 6.0 Hz), 1.31–1.60 (5H, m), 1.68–1.82 (2H, m), 1.87–2.08 (4H, m), 2.14–2.20 (1H, m), 2.36–2.40 (1H, m), 3.11 (1H, m), 3.29 (1H, br), 3.34 (1H, td, *J* = 9.2, 3.6 Hz); ^13^C-NMR (100 MHz CDCl_3_) δ: 13.84, 15.11, 19.01, 23.91, 25.14, 27.48, 28.89, 35.47, 36.00, 39.18, 57.06, 59.69, 74.85; IR (neat): 1375, 1456, 1636, 1647, 2866, 2926, 2955, 3647 cm^−1^; MS (EI) *m*/*z* 211 (M^+^); HRMS (EI) Calcd for C_13_H_25_NO: 211.1936 (M^+^); Found 211.1928; [α]_D_^20^ −5.3 (*c* 0.4, CHCl_3_)

### 3.2. Electrophysiological Recording of Nicotinic ACh Receptor-Mediated Current in Xenopus Oocytes

*Xenopus* oocytes expressing recombinant mouse α7- and α4β2-nicotinic ACh receptors were prepared by injection of the plasmid containing respective subunit cDNAs (provided by Dr. J. A. Stitzel, University of Michigan Medical Center), according to the protocols described previously [29]. The oocytes were cultured at 19 °C for 3 to 6 days in 50% Leibovitz’s L-15 Medium (11415064, Thermo Fisher Scientific, MA, USA, pH 7.5) containing 1 μg/mL insulin and 100 μg/mL gentamicin (078-06061, Fujifilm Wako Pure Chemical Corp., Osaka, Japan). Two-electrode voltage-clamp recordings were then performed, as described previously [29]. In brief, an oocyte was placed in a 300 μL tube-like chamber where Ringer solution (82.5 mM NaCl, 2.5 mM KCl, 2.5 mM CaCl_2_, 1 mM MgCl_2_, and 5 mM HEPES, pH 7.4) containing 1 μM atropine (A0257, Sigma–Aldrich, MA, USA) was perfused at a rate of 15 mL/min. Membrane potential was held at −60 mV, and currents were measured using a GeneClamp 500 amplifier, Digidata1322A, and pClamp9 software (Axon Instruments, Union City, CA, USA). The oocyte was pretreated with an alkaloid for 3 min and then treated with ACh (011-00592, Fujifilm Wako Pure Chemical) for 5 s. Each solution was rapidly switched by using a three-way valve (AG41-02-2-D-AC100V Multilex Valve, CKD Corp., Aichi, Japan). To analyze the effects of alkaloids, current response to ACh in the presence of alkaloid was normalized to control response (i.e., current elicited by ACh (100 μM for α7 and 1 μM for α4β2) alone)) in each oocyte and then averaged.

### 3.3. Ligand-Binding Assays

The [^3^H]nicotine and [^3^H]methyllycaconitine binding assays using membrane suspensions from whole rat brain (excluding cortex and cerebellum) were performed as described previously [50].

### 3.4. In Vitro Effect of the Compound on the Transport of Cationic Compounds at the BBB and Inner BRB

As model cells of the rat BBB and inner BRB, TR-BBB and TR-iBRB2 cells were utilized [48,49]. These cells were cultured following the previous manuscript [51] and seeded onto collagen type I-coated 24-well plate (Corning, Kennebunk, ME, USA) at a density of 5 × 10^4^ cells/cm^2^. The cells were cultured for 2 days and then washed with extracellular fluid buffer (122 mM NaCl, 25 mM NaHCO_3_, 3 mM KCl, 1.4 mM CaCl_2_, 1.2 mM MgSO_4_, 0.4 mM K_2_HPO_4_, 10 mM D-glucose, 10 mM 2-[4-(2-hydroxyethyl)-1-piperazinyl]ethanesulfonic acid-NaOH, pH 7.4) at 37 °C. The extracellular fluid buffer containing [^3^H]nicotine (85 Ci/mmol; American Radiolabeled Chemicals, St. Louis, MO, USA) or [^3^H]verapamil (80 Ci/mmol; American Radiolabeled Chemicals) at a concentration of 0.5 μCi/mL in the absence or presence of test compounds at 200 µM with 1.0% DMSO was applied to the cells and incubated at 37 °C for designed time ([^3^H]nicotine, 10 sec; [^3^H]verapamil, 3 min). After the uptake reaction, the cells were rinsed with the extracellular fluid buffer at 4 °C and solubilized in 1 N NaOH. The solubilized solution was neutralized with 1 N HCl. The ^3^H-radioactivities derived from the cell-solubilized solution and reaction buffer were measured using an AccuFLEX LSC-7400 liquid scintillation counter (Hitachi, Kashiwa, Japan). Protein concentration in the solubilized solution was quantified by a DC protein assay kit II (BIO-RAD, Hercules, CA, USA). The uptake activities were normalized by the concentration of the radiolabeled compound in the transport buffer and the cellular protein amount in each well. The data were expressed as the percentage of uptake activity in the control group and mean ± S.E.M. Statistic difference was evaluated using a one-way analysis of variance followed by Dunnett’s test.

## Figures and Tables

**Table 1:** Inhibitory effects of ent-*cis*-**195A** and *cis*-**211A** on [^3^H]nicotine uptake by TR-BBB13 cells and [^3^H]verapamil uptake by TR-iBRB2 cells.

Conditions	Percentage of Control	
	[^3^H]Nicotine Uptake	[^3^H]Verapamil Uptake
Control	100 ± 5	100 ± 4
ent-*cis*-**195A**	31.6 ± 1.7 *	66.9 ± 2.5 *
*cis*-**211A**	60.0 ± 3.5 *	70.6 ± 4.1 *

[^3^H]Nicotine uptake (0.1 μCi/well, 6.0 nM) by TR-BBB13 cells was measured at 37 °C for 10 sec in the absence (control) or presence of test compounds at 200 µM with 1.0% dimethyl sulfoxide (DMSO). Similarly, [^3^H]verapamil uptake by TR-iBRB2 cells was performed at 37 °C for 3 min. Each value represents the mean ± standard error of the mean S.E.M. (*n* = 3–6). *p < 0.01, significantly different from the control in Dunnett’s test.

## Supplementary Materials

The NMR spectra (^1^H-NMR, ^13^C-NMR) of all new compounds are available online.

## Sample Availability

Samples of *cis*-**195A** and *cis*-**211A** are available from the authors.
