# Supplementary material for: Total Synthesis of Decahydroquinoline Poison Frog Alkaloids ent-cis-195A and cis-211A
Source: Molecules. 2021 Dec 12;26(24):7529. doi: 10.3390/molecules26247529 (PMC8706607; doi:10.3390/molecules26247529)
Supplement: Supplementary file 1 [file molecules-26-07529-s001.zip › molecules-1493580-supplementary.pdf]

Supplementary Materials

Total Synthesis of Decahydroquinoline Poison Frog Alkaloids ent-*cis*-**195A** and *cis*-**211A**

Takuya Okada [1,†,*], Naizhen Wu [2,†], Katsuki Takashima [3], Jungoh Ishimura [3], Hiroyuki Morita [4], Takuya Ito [4,5], Takeshi Kodama [4], Yuhei Yamasaki [2], Shin-ichi Akanuma [2], Yoshiyuki Kubo [2], Ken-ichi Hosoya [2], Hiroshi Tsuneki [2], Tsutomu Wada [2], Toshiyasu Sasaoka [2], Takahiro Shimizu [2], Hideki Sakai [2], Linda P. Dwoskin [6], Syed R. Hussaini [7], Ralph A. Saporito [8], and Naoki Toyooka [1,3,#,*]

[1] Graduate School of Innovative Life Science, University of Toyama, 3190 Gofuku, Toyama 930-8555, Japan

[2] Graduate School of Medicine and Pharmaceutical Sciences, University of Toyama, 2630 Sugitani, Toyama 930-0194, Japan

[3] Graduate School of Science and Engineering, University of Toyama, 3190 Gofuku, Toyama 930-8555, Japan

[4] Institute of Natural Medicine, University of Toyama, 2630 Sugitani, Toyama 930-0194, Japan

[5] Faculty of Pharmacy, Osaka Ohtani University, Tondabayashi, Osaka 584-8540, Japan

[6] Department of Pharmaceutical Sciences, College of Pharmacy, University of Kentucky, Lexington, KY 40536, USA

[7] Department of Chemistry and Biochemistry, The University of Tulsa, 800 S. Tucker Dr., Tulsa, OK 74104, USA

[8] Department of Biology, John Carroll University, University Heights, Ohio 44118, USA

[†] These authors equally contributed to this study.

* Correspondence: tokada@eng.u-toyama.ac.jp (T.O.); toyooka@eng.u-toyama.ac.jp (N. T.); Tel.: +81-76-445-6859 (N.T.)

[#] Dedicated to my honorable Professor Daniel Comins for his outstanding contributions to heterocyclic and organometallic chemistry.

Table of Contents

1. Copies of ^{1}H- and ^{13}C-NMR Spectra

1. Copies of ^{1}H- and ^{13}C-NMR Spectra

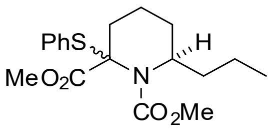

3

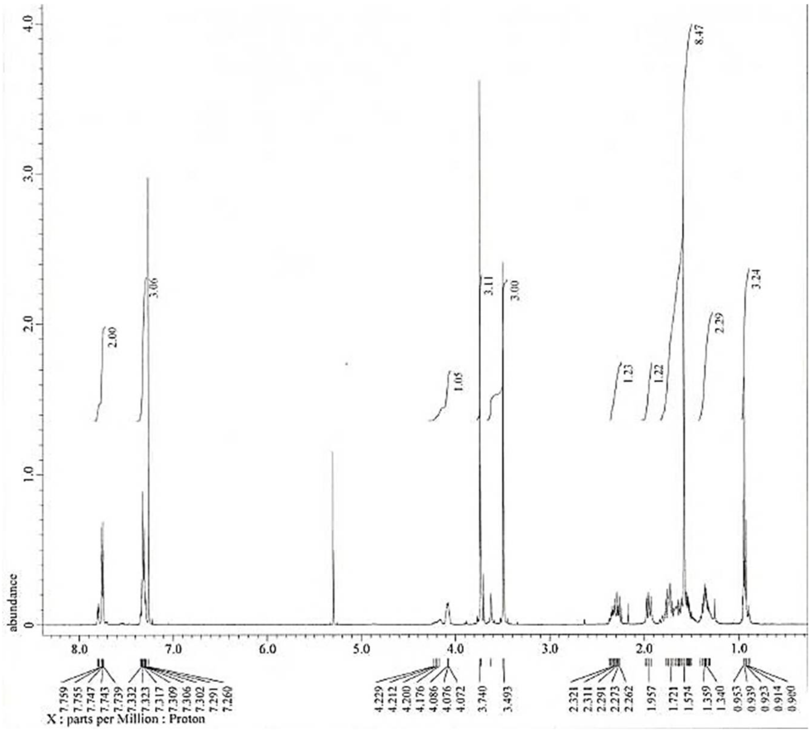

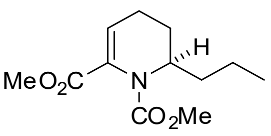
MeO2C
N
H
CO2Me

4

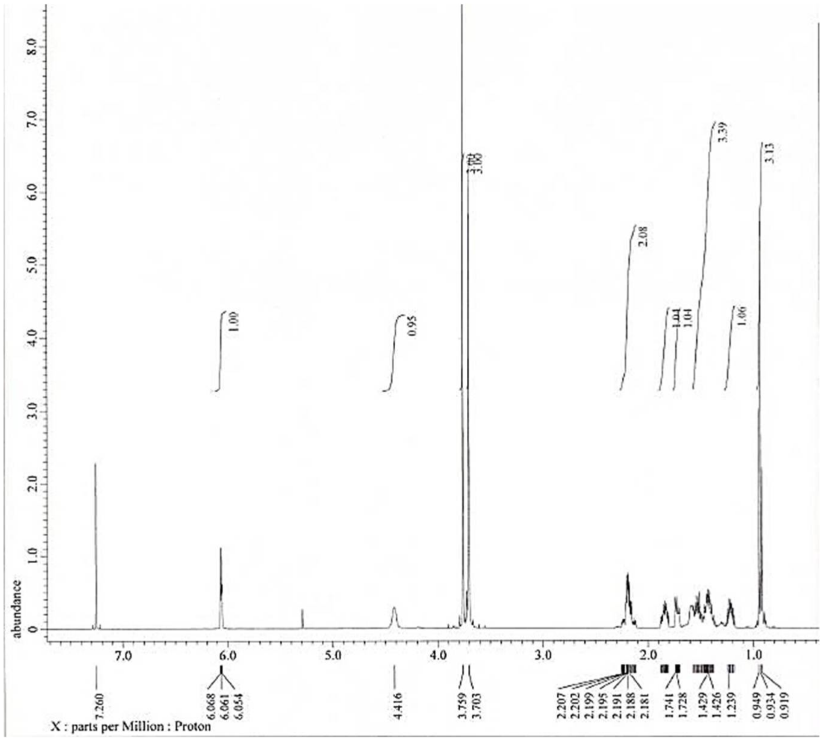
abundance
X : parts per Million : Proton
1.00
0.95
3.03
3.00
2.08
1.01
1.04
3.39
1.06
3.13
7.260
6.068
6.061
6.054
4.416
3.759
3.703
2.207
2.202
2.199
2.195
2.191
2.188
2.181
1.741
1.728
1.429
1.426
1.239
0.949
0.934
0.919

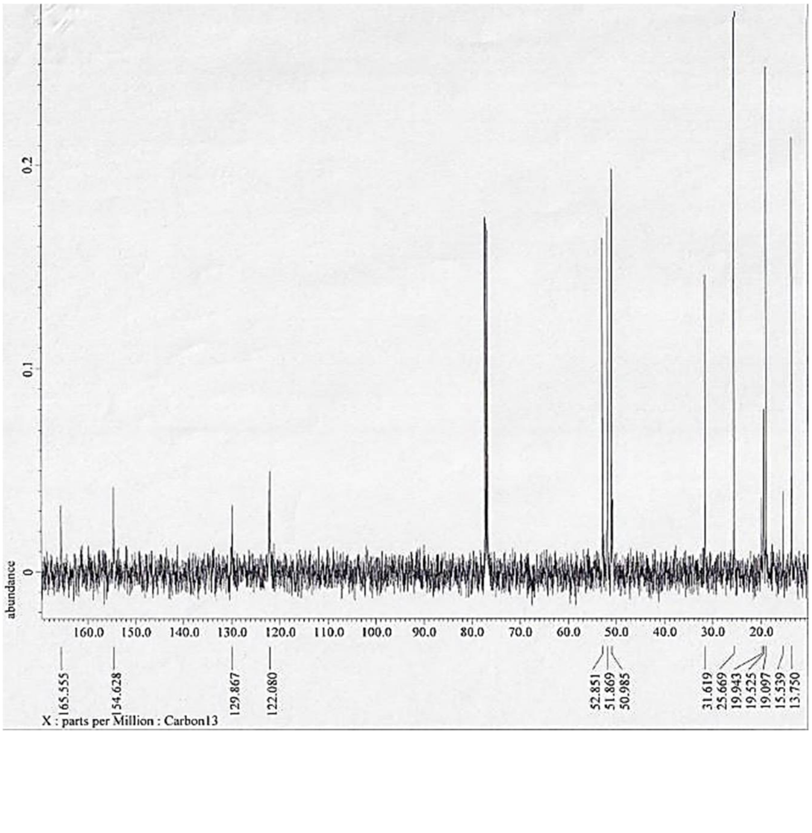
abundance
X : parts per Million : Carbon13
165.555
154.628
129.867
122.080
52.851
51.869
50.985
31.619
25.669
19.943
19.525
19.097
15.539
13.750

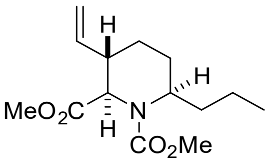
MeO2C
N
CO2Me
H
H
H

5

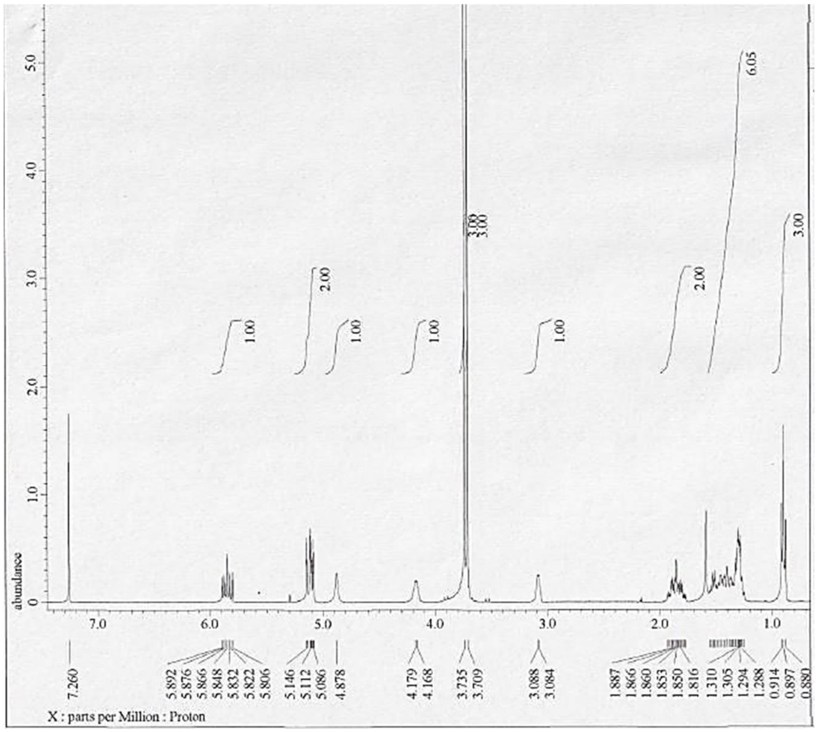
abundance
X : parts per Million : Proton

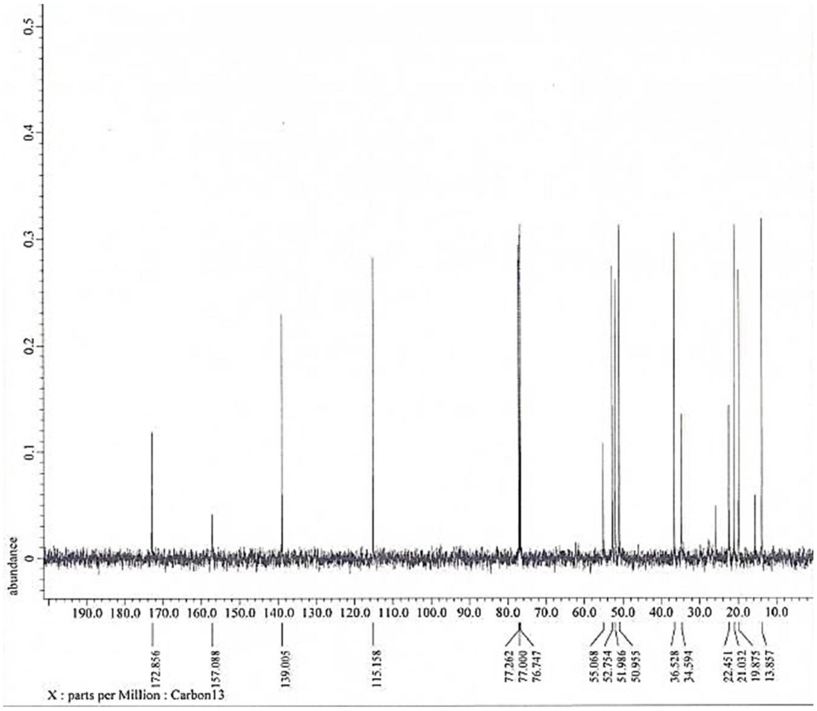
abundance
X : parts per Million : Carbon13

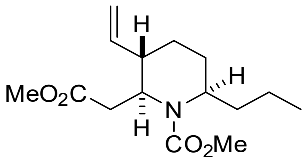
MeO2C
H
H
H
N
CO2Me

6

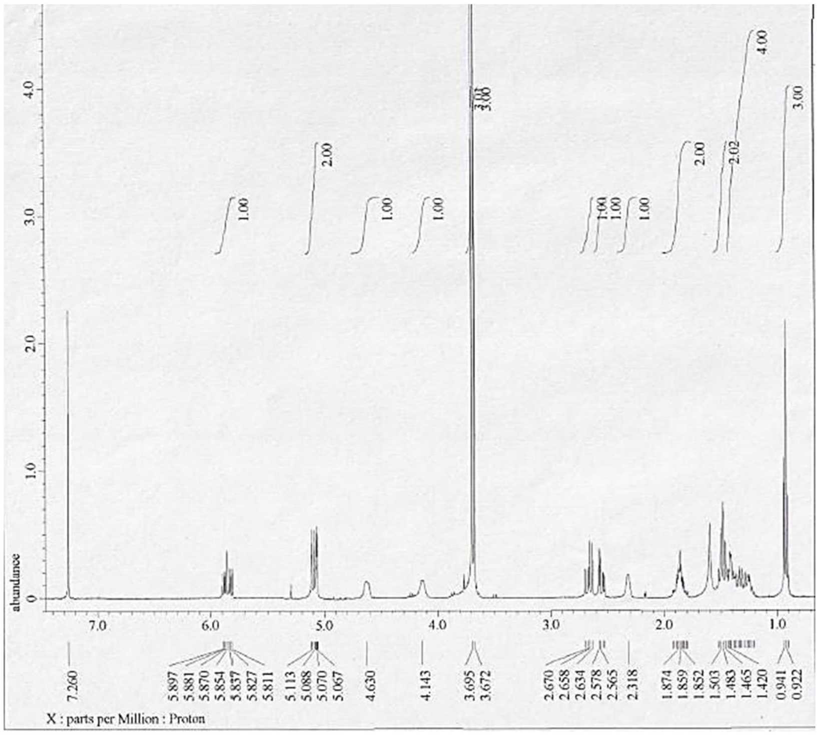
abundance
X : parts per Million : Proton
7.260
5.897
5.881
5.870
5.854
5.837
5.827
5.811
5.113
5.088
5.070
5.067
4.630
4.143
3.695
3.672
2.670
2.658
2.634
2.578
2.565
2.318
1.874
1.859
1.852
1.503
1.483
1.465
1.420
0.941
0.922

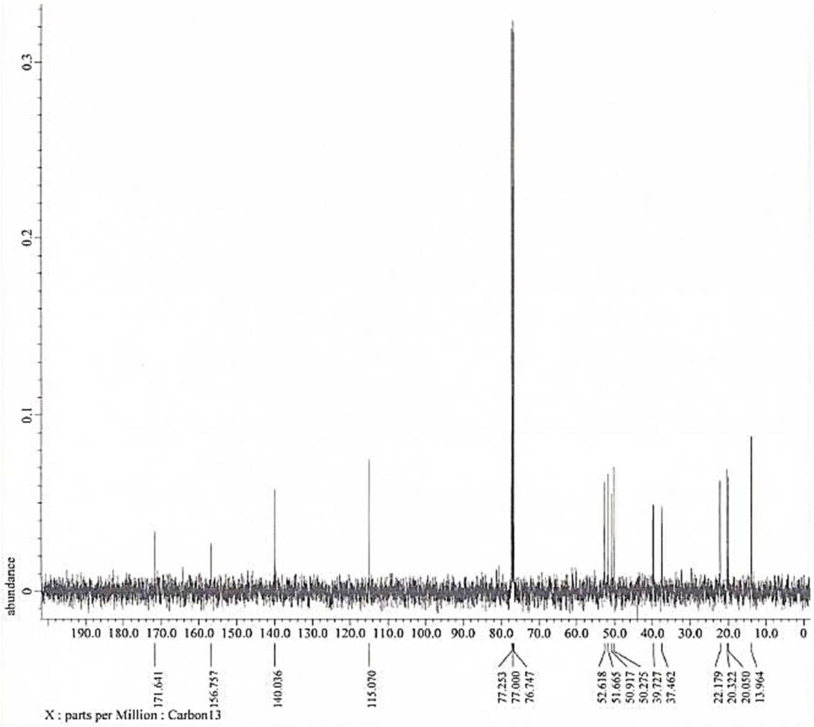
abundance
X : parts per Million : Carbon13
171.641
156.757
140.036
115.070
77.253
77.000
76.747
52.618
51.665
50.917
50.275
39.727
37.462
22.179
20.322
20.050
13.964

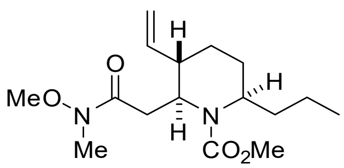
MeO
N
Me
O
H
H
N
CO2Me
H

7

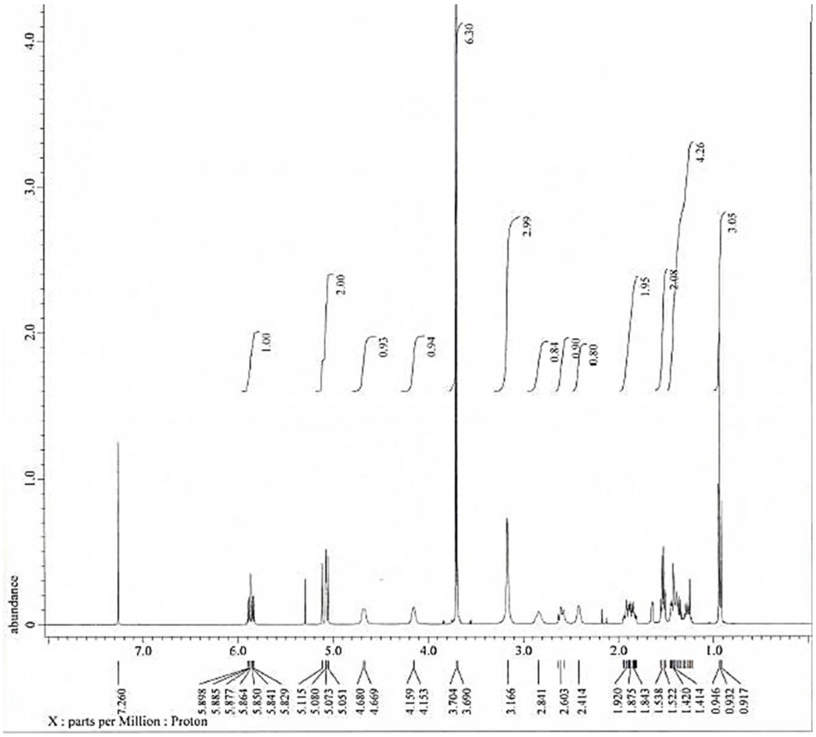
abundance
X : parts per Million : Proton
7.260
5.898 5.885 5.877 5.864 5.850 5.841 5.829
5.115 5.080 5.073 5.051
4.680 4.669
4.159 4.153
3.704 3.690
3.166
2.841
2.603
2.414
1.920 1.875 1.843 1.538 1.522 1.420 1.414
0.946 0.932 0.917
1.00
2.00
0.93
0.94
6.30
2.99
0.84
0.90
0.80
1.95
2.08
4.26
3.05

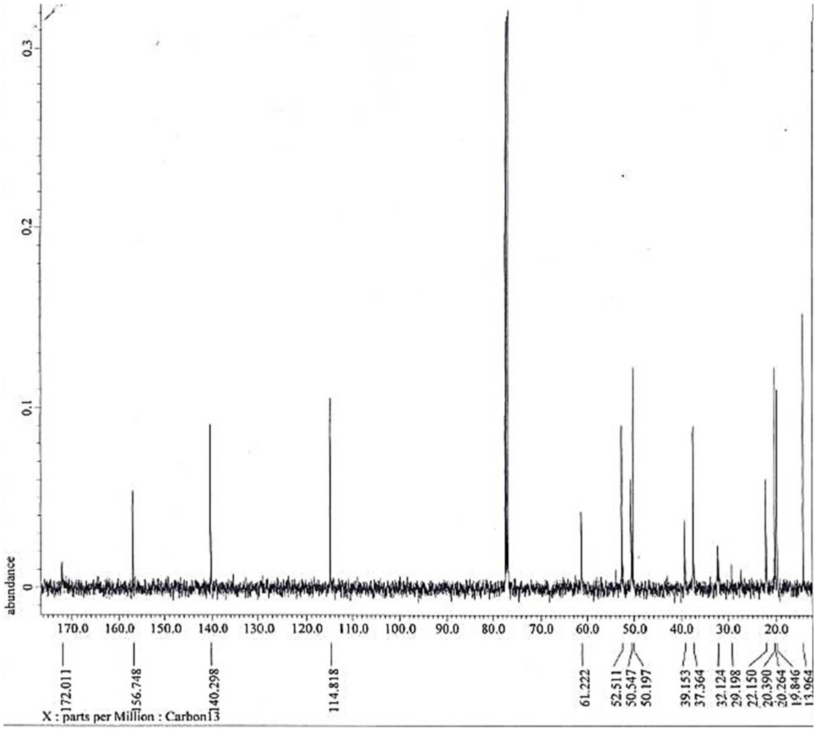
abundance
X : parts per Million : Carbon13
172.011
156.748
140.298
114.818
61.222
52.511
50.547
50.197
39.153
37.364
32.124
29.198
22.150
20.390
20.264
19.846
13.964

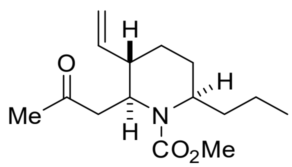

O
H
H
Me
H
N
CO2Me

8

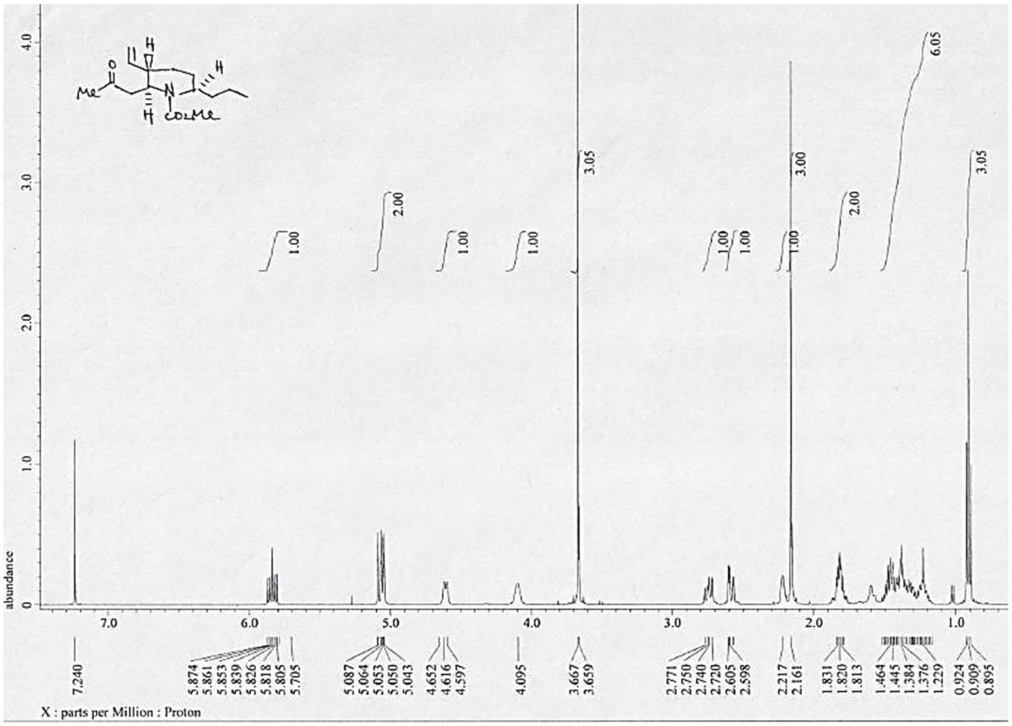

abundance
1.00
2.00
1.00
1.00
3.05
1.00
1.00
1.00
3.00
2.00
6.05
3.05
7.240
5.874
5.861
5.853
5.839
5.826
5.818
5.805
5.705
5.087
5.064
5.053
5.050
5.043
4.652
4.616
4.597
4.095
3.667
3.659
2.771
2.750
2.740
2.720
2.605
2.598
2.217
2.161
1.831
1.820
1.813
1.464
1.445
1.384
1.376
1.229
0.924
0.909
0.895
X : parts per Million : Proton

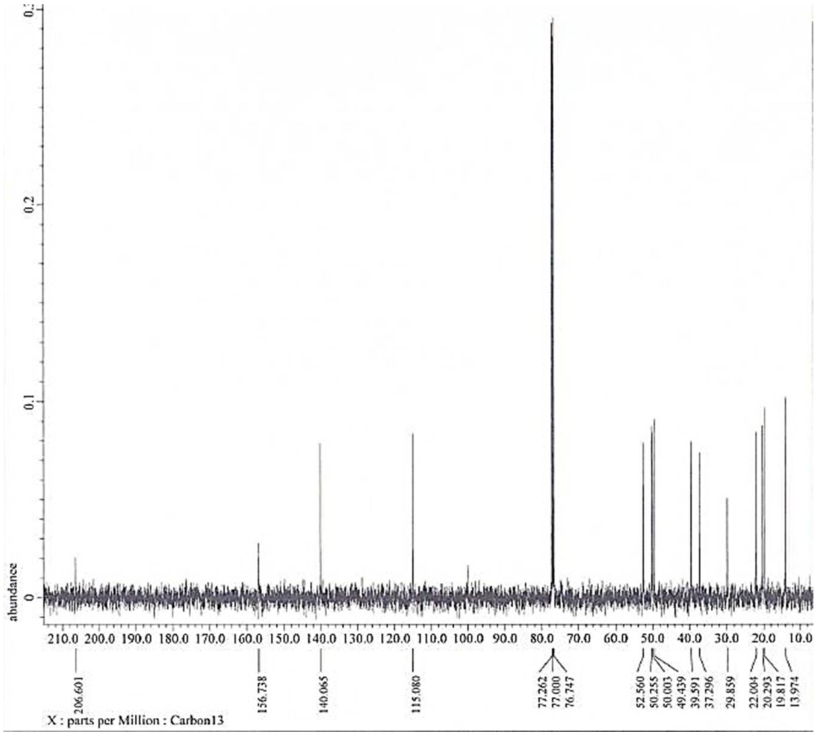

abundance
206.601
156.738
140.065
115.080
77.262
77.000
76.747
52.560
50.255
50.003
49.439
39.591
37.296
29.859
22.004
20.293
19.817
13.974
X : parts per Million : Carbon13

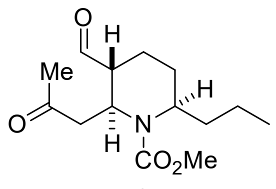
Me
O
H
H
N
H
CO2Me

9

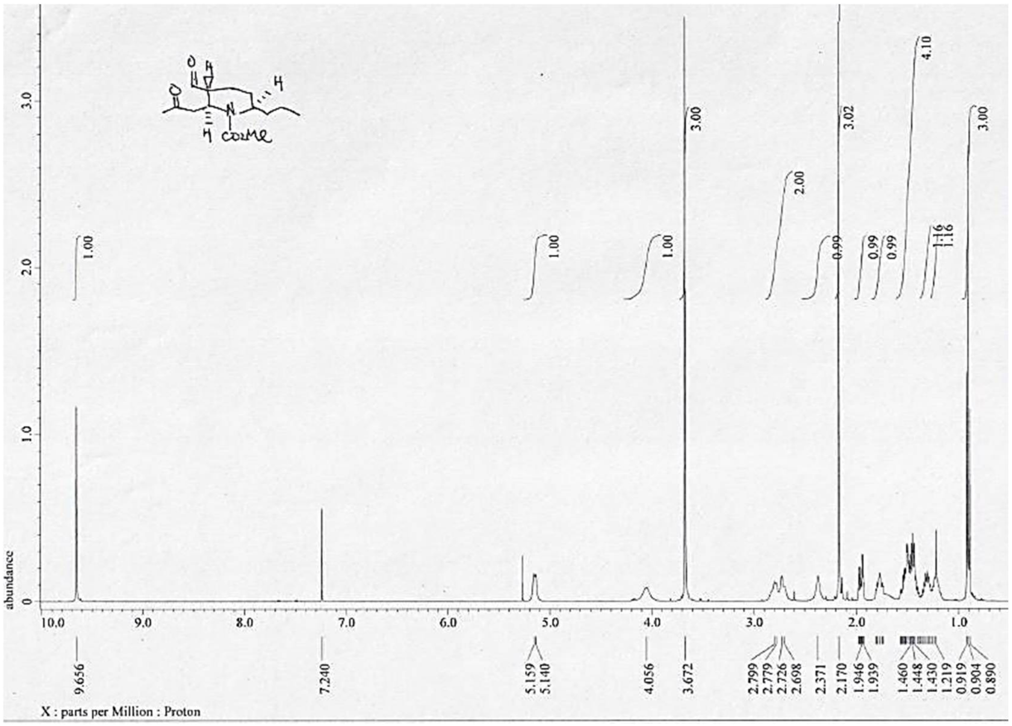
abundance
X : parts per Million : Proton
9.656
7.240
5.159
5.140
4.056
3.672
2.799
2.779
2.726
2.698
2.371
2.170
1.946
1.939
1.460
1.448
1.430
1.219
0.919
0.904
0.890
1.00
1.00
1.00
3.00
2.00
0.99
3.02
0.99
0.99
4.10
1.16
1.16
3.00

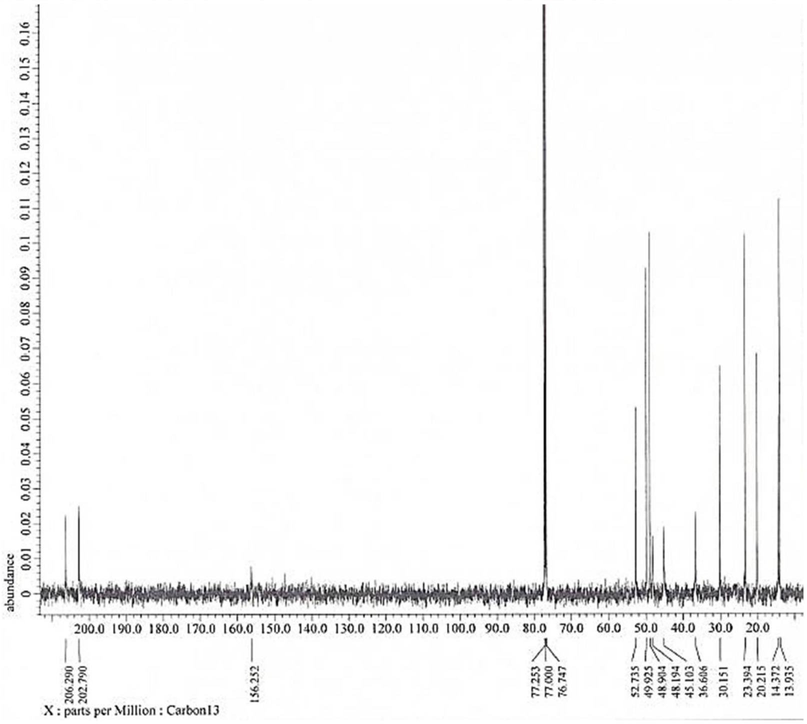
abundance
X : parts per Million : Carbon13
206.290
202.790
156.252
77.253
77.000
76.747
52.735
49.925
48.904
48.194
45.103
36.686
30.151
23.394
20.215
14.372
13.935

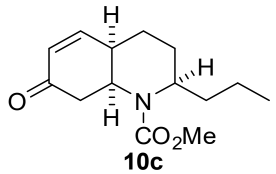

10c

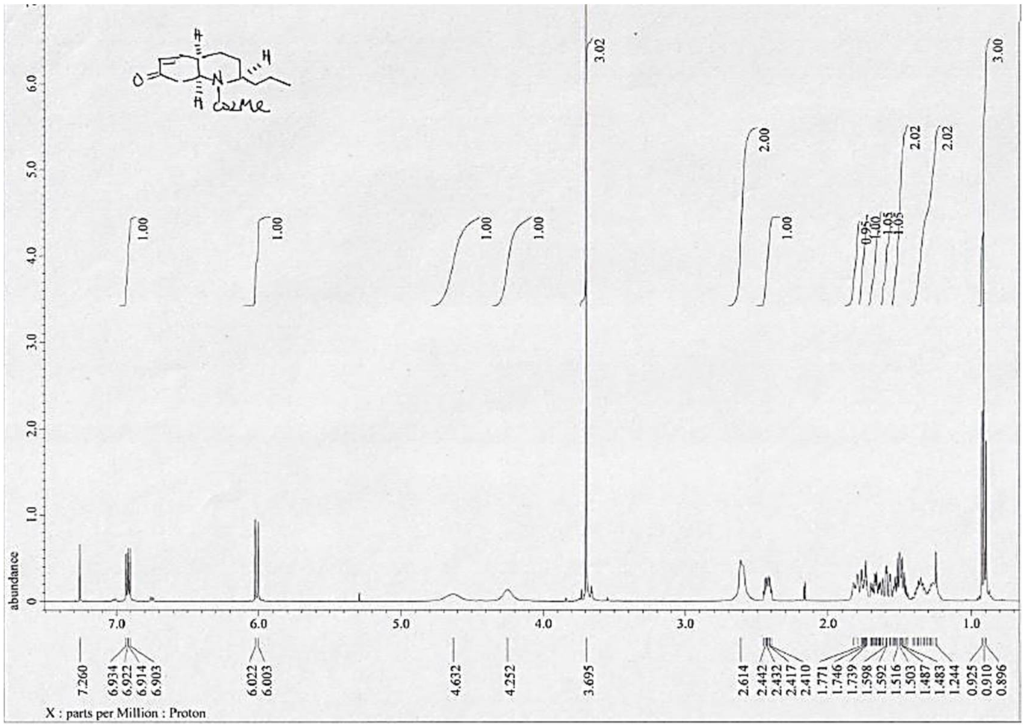

3.02
3.00
2.00
2.02
2.02
1.00
1.00
1.00
1.00
1.00
abundance
7.260
6.934
6.922
6.914
6.903
6.022
6.003
4.632
4.252
3.695
2.614
2.442
2.432
2.417
2.410
1.771
1.746
1.739
1.598
1.592
1.516
1.503
1.487
1.483
1.244
0.925
0.910
0.896
X : parts per Million : Proton

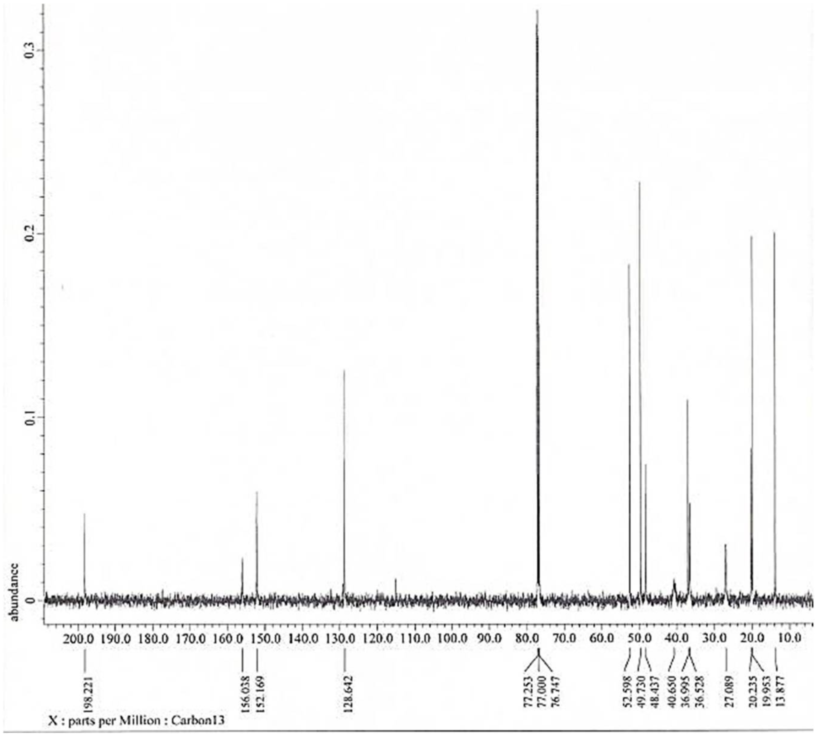

abundance
198.221
156.038
152.169
128.642
77.253
77.000
76.747
52.598
49.730
48.437
40.650
36.995
36.528
27.089
20.235
19.953
13.877
X : parts per Million : Carbon13

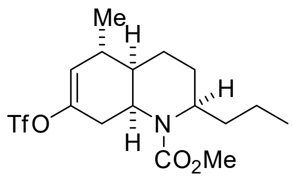
Me
H
H
TfO
N
H
CO2Me

11

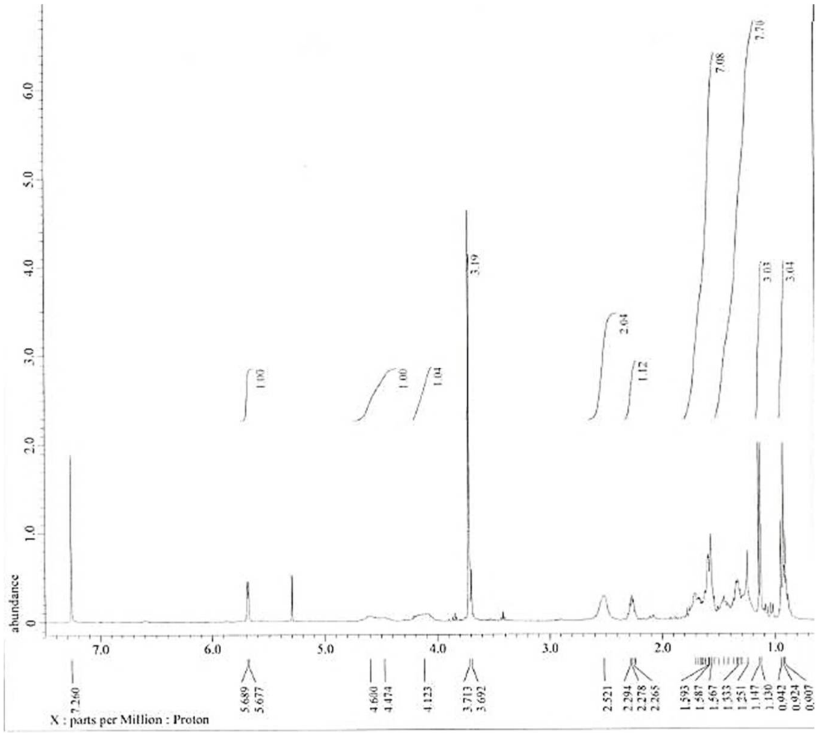
abundance
1.00
1.00
1.04
3.19
2.04
1.12
7.08
7.70
3.03
3.04
7.260
5.689
5.677
4.680
4.474
4.123
3.713
3.692
2.521
2.294
2.278
2.265
1.593
1.587
1.567
1.533
1.251
1.147
1.130
0.942
0.924
0.907
X : parts per Million : Proton

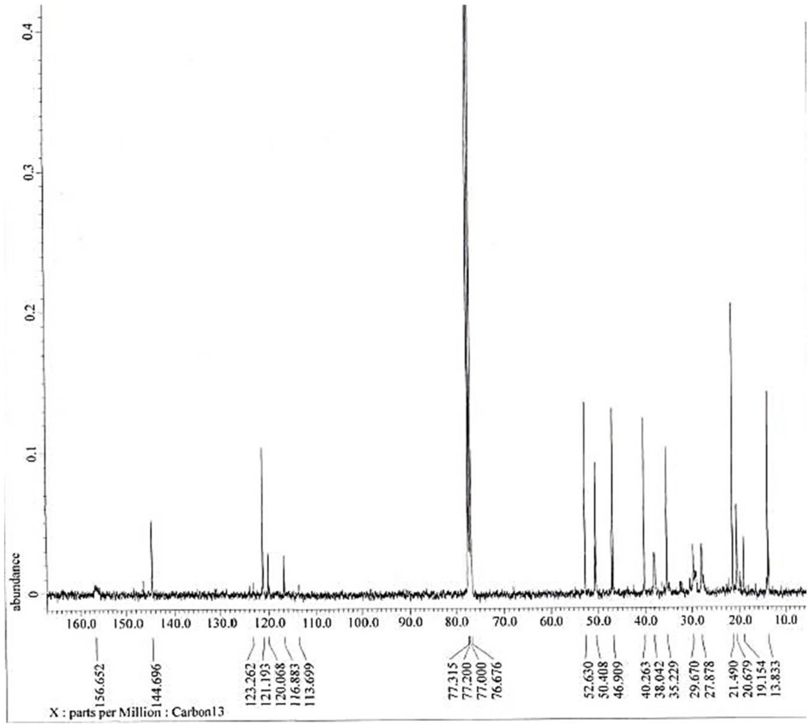
abundance
156.652
144.696
123.262
121.193
120.068
116.883
113.699
77.315
77.200
77.000
76.676
52.630
50.408
46.909
40.263
38.042
35.229
29.670
27.878
21.490
20.679
19.154
13.833
X : parts per Million : Carbon13

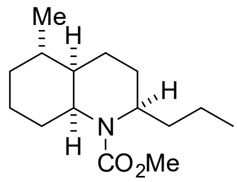
Me
H
H
N
H
CO_2Me

12

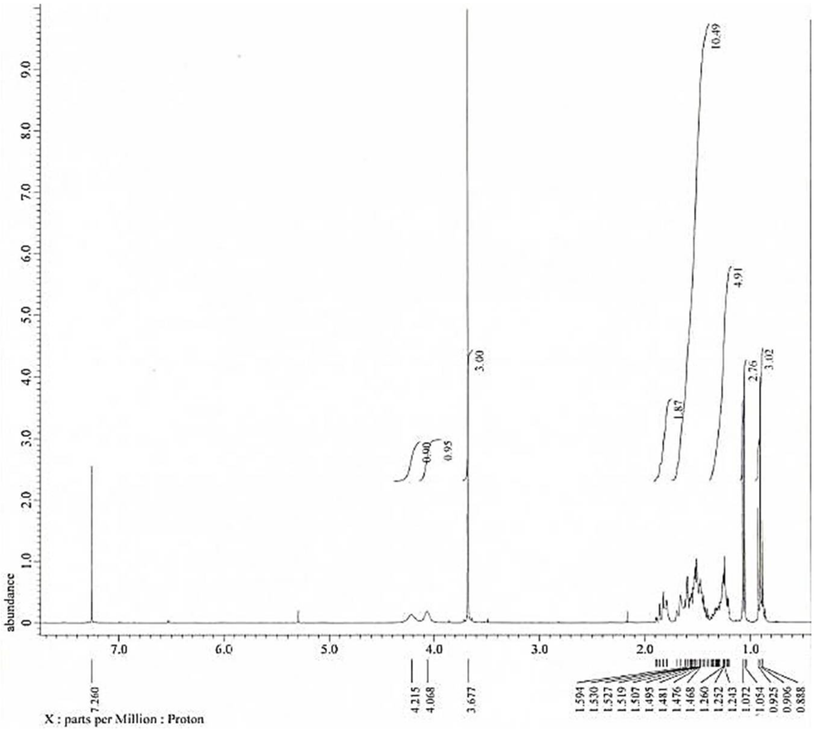
abundance
10.49
4.91
3.00
1.87
0.90
0.95
2.76
3.02
7.260
4.215
4.068
3.677
1.594
1.530
1.527
1.519
1.507
1.495
1.481
1.476
1.468
1.260
1.252
1.243
1.072
1.054
0.925
0.906
0.888
X : parts per Million : Proton

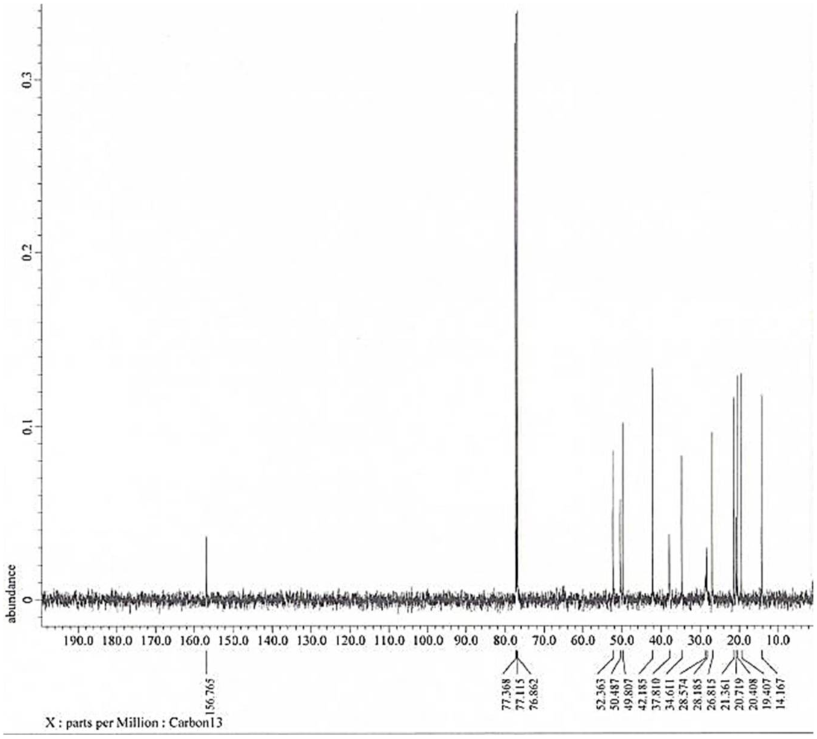
abundance
156.765
77.368
77.115
76.862
52.363
50.487
49.807
42.185
37.810
34.611
28.574
28.185
26.815
21.361
20.719
20.408
19.407
14.167
X : parts per Million : Carbon13

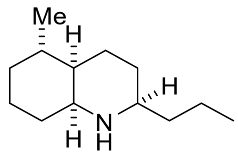
Me
H
H
N
H
H

ent-*cis*-**195A**

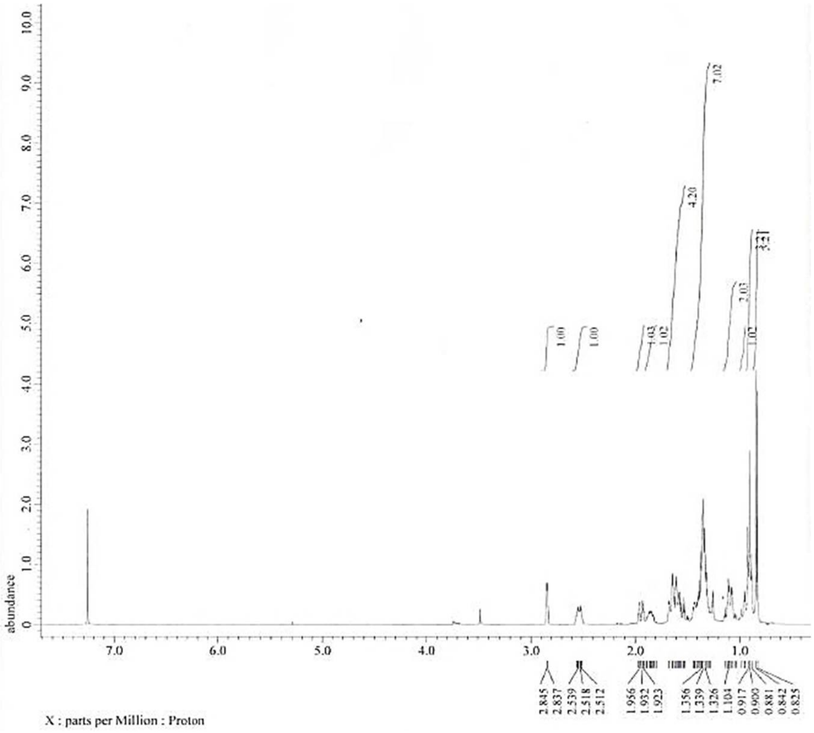
abundance
1.00
1.00
1.03
1.02
4.20
7.02
2.03
1.02
3.21
2.845
2.837
2.539
2.518
2.512
1.956
1.932
1.923
1.356
1.339
1.326
1.104
0.917
0.900
0.881
0.842
0.825
X : parts per Million : Proton

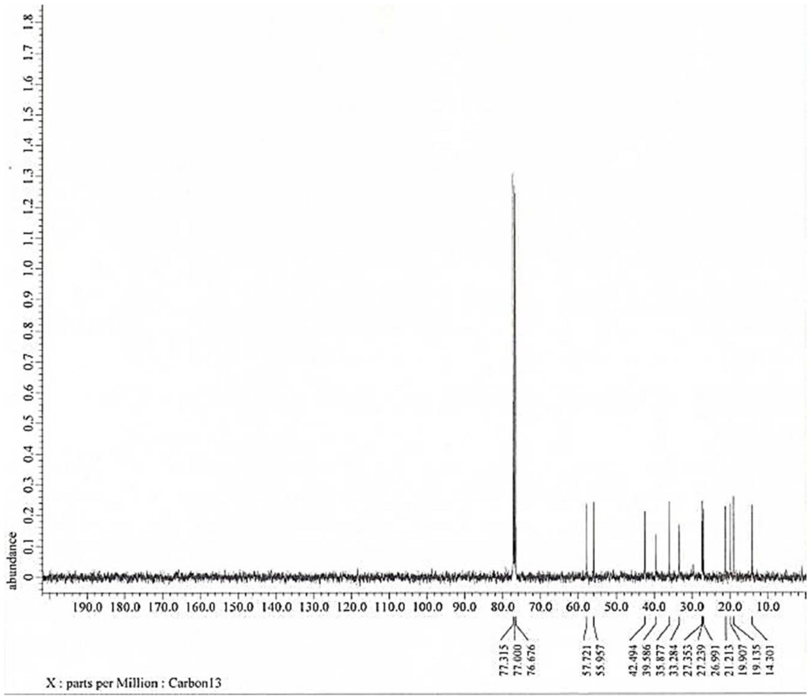
abundance
77.315
77.000
76.676
57.721
55.957
42.494
39.586
35.877
33.284
27.353
27.239
26.991
21.213
19.907
19.135
14.301
X : parts per Million : Carbon13

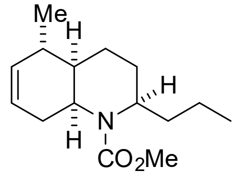
Me
H
H
N
H
CO2Me

13

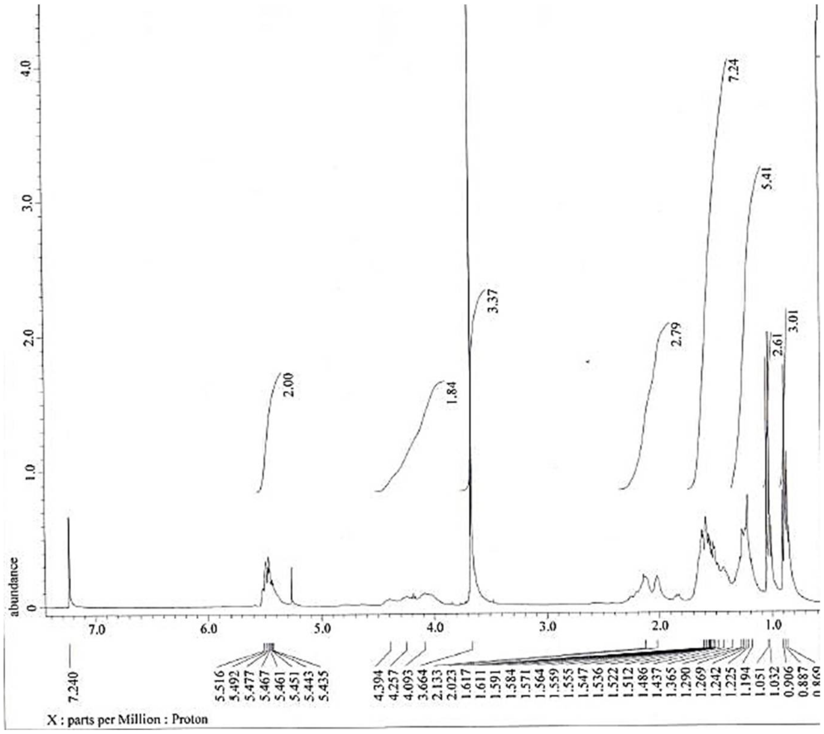
abundance
0
1.0
2.0
3.0
4.0
7.0
6.0
5.0
4.0
3.0
2.0
1.0
2.00
1.84
3.37
2.79
7.24
5.41
2.61
3.01
7.240
5.516
5.492
5.477
5.467
5.461
5.451
5.443
5.435
4.394
4.257
4.093
3.664
2.133
2.023
1.617
1.611
1.591
1.584
1.571
1.564
1.559
1.555
1.547
1.536
1.522
1.512
1.486
1.437
1.365
1.290
1.269
1.242
1.225
1.194
1.051
1.032
0.906
0.887
X : parts per Million : Proton

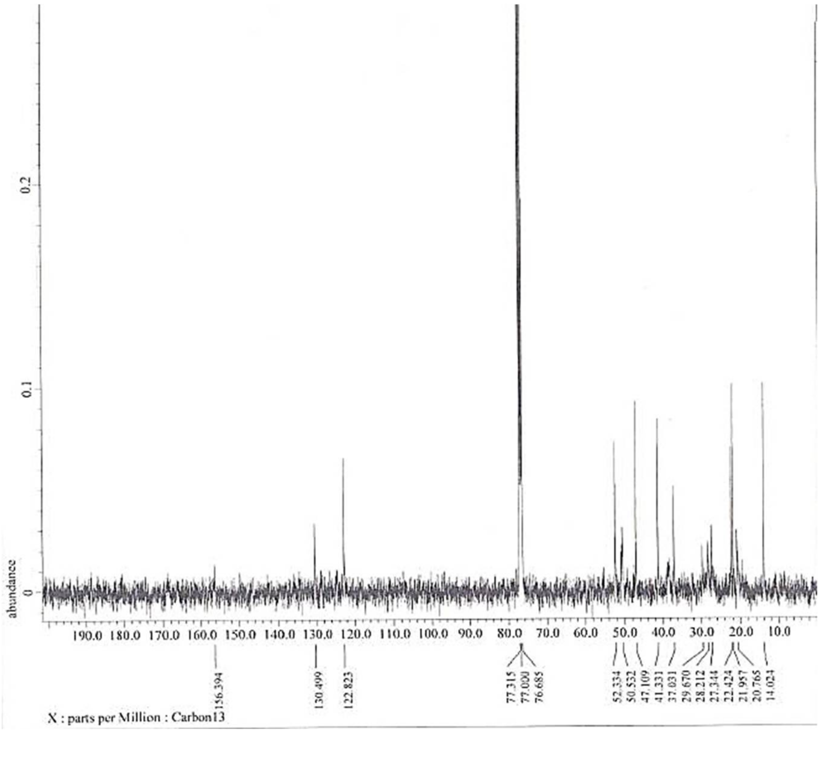
abundance
0
0.1
0.2
190.0
180.0
170.0
160.0
150.0
140.0
130.0
120.0
110.0
100.0
90.0
80.0
70.0
60.0
50.0
40.0
30.0
20.0
10.0
156.394
130.499
122.823
77.315
77.000
76.685
52.334
50.532
47.109
41.331
37.031
29.670
28.212
27.344
22.424
21.957
20.765
14.024
X : parts per Million : Carbon13

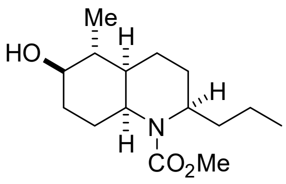
Me
H
HO
H
N
H
CO2Me

15

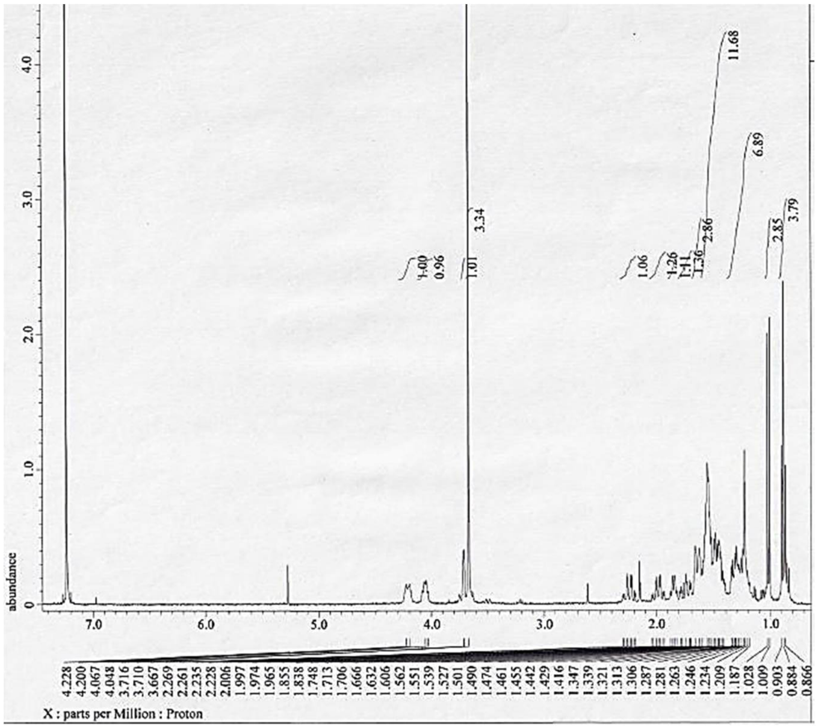
abundance
X : parts per Million : Proton
1.00
0.96
1.01
3.34
1.06
1.26
1.11
1.36
2.86
11.68
6.89
2.85
3.79
4.228
4.200
4.067
4.048
3.716
3.710
3.667
2.269
2.261
2.235
2.228
2.006
1.997
1.974
1.965
1.855
1.838
1.748
1.713
1.706
1.666
1.632
1.606
1.562
1.551
1.539
1.527
1.501
1.490
1.474
1.461
1.455
1.442
1.429
1.416
1.347
1.339
1.321
1.313
1.306
1.287
1.281
1.263
1.246
1.234
1.209
1.187
1.028
1.009
0.903
0.884
0.866

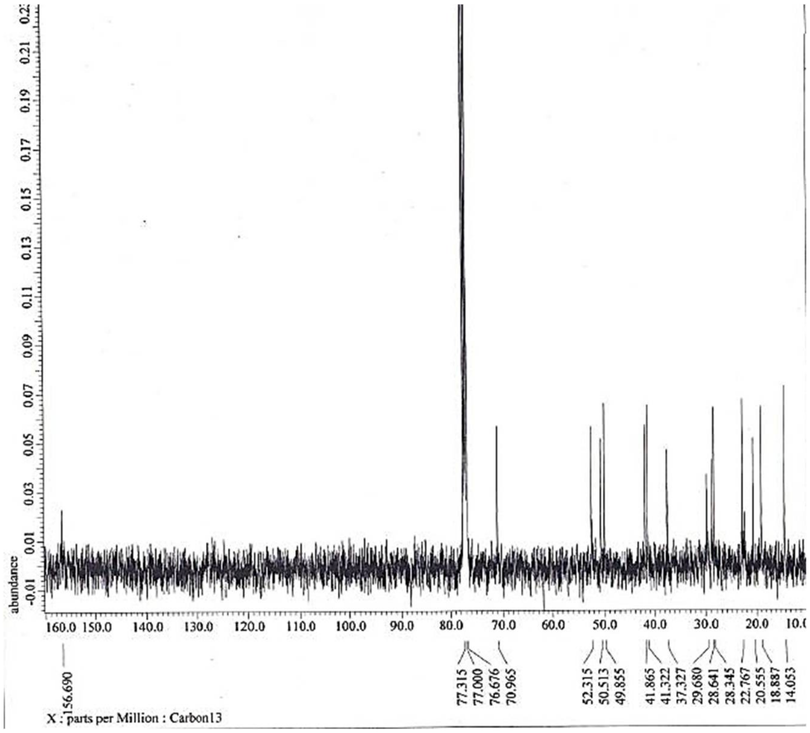
abundance
X : parts per Million : Carbon13
156.690
77.315
77.000
76.676
70.965
52.315
50.513
49.855
41.865
41.322
37.327
29.680
28.641
28.345
22.767
20.555
18.887
14.053

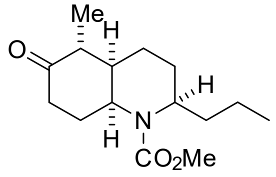

Me
H
O
H
N
H
CO2Me

19

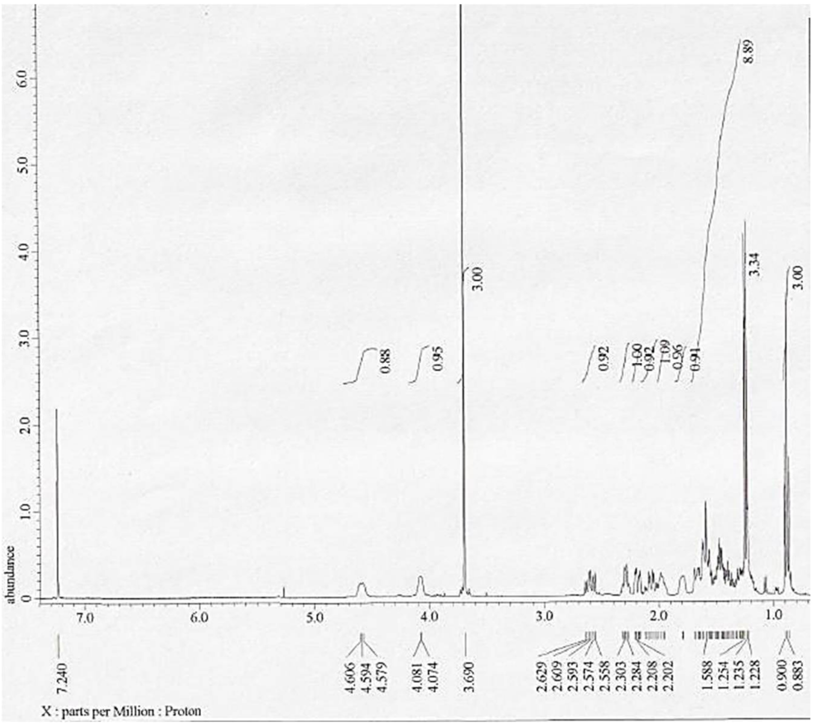

abundance
0.88
0.95
3.00
0.92
1.00
0.92
1.99
0.96
0.91
8.89
3.34
3.00
7.240
4.606
4.594
4.579
4.081
4.074
3.690
2.629
2.609
2.593
2.574
2.558
2.303
2.284
2.208
2.202
1.588
1.254
1.235
1.228
0.900
0.883
X : parts per Million : Proton

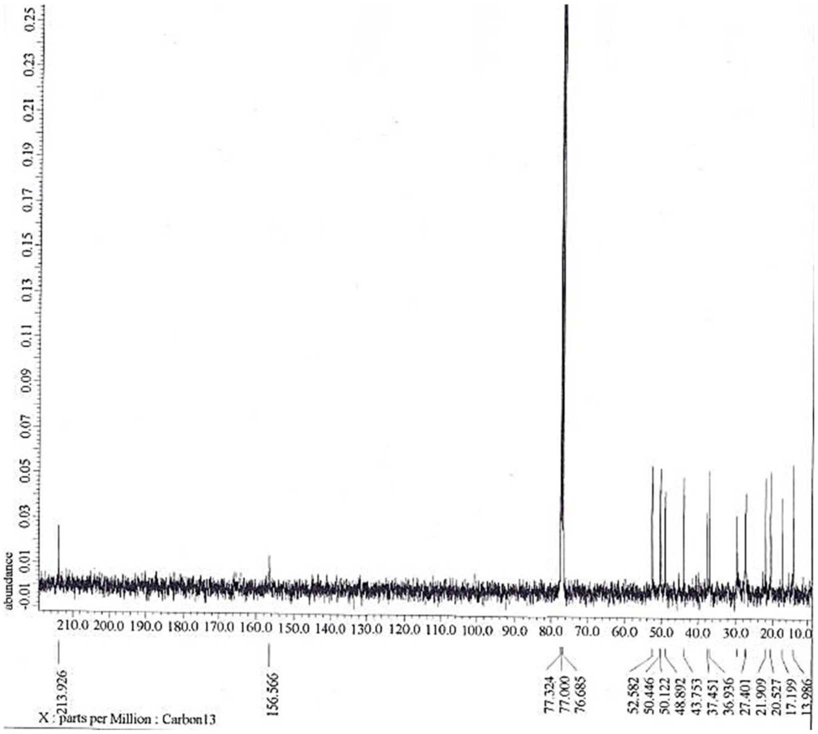

abundance
213.926
156.566
77.324
77.000
76.685
52.582
50.446
50.122
48.892
43.753
37.451
36.936
27.401
21.909
20.527
17.199
13.986
X : parts per Million : Carbon13

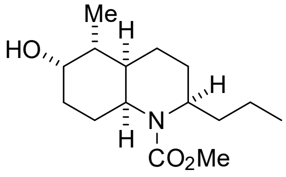
Me
H
HO
H
H
N
CO2Me

16

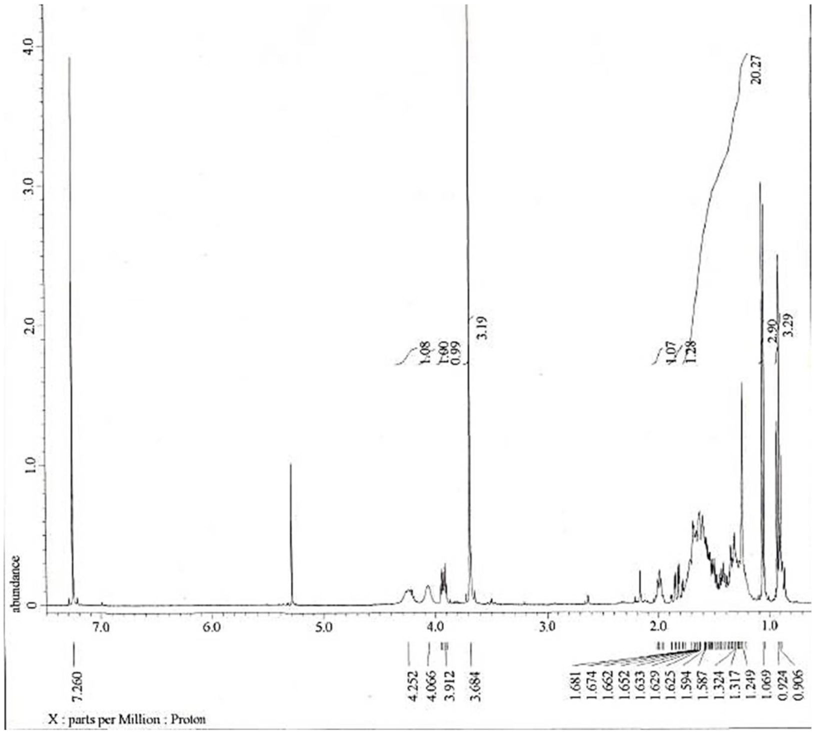
abundance
X : parts per Million : Proton
1.08
1.00
0.99
3.19
1.07
1.28
20.27
2.90
3.29
7.260
4.252
4.066
3.912
3.684
1.681
1.674
1.662
1.652
1.633
1.629
1.625
1.594
1.587
1.324
1.317
1.249
1.069
0.924
0.906

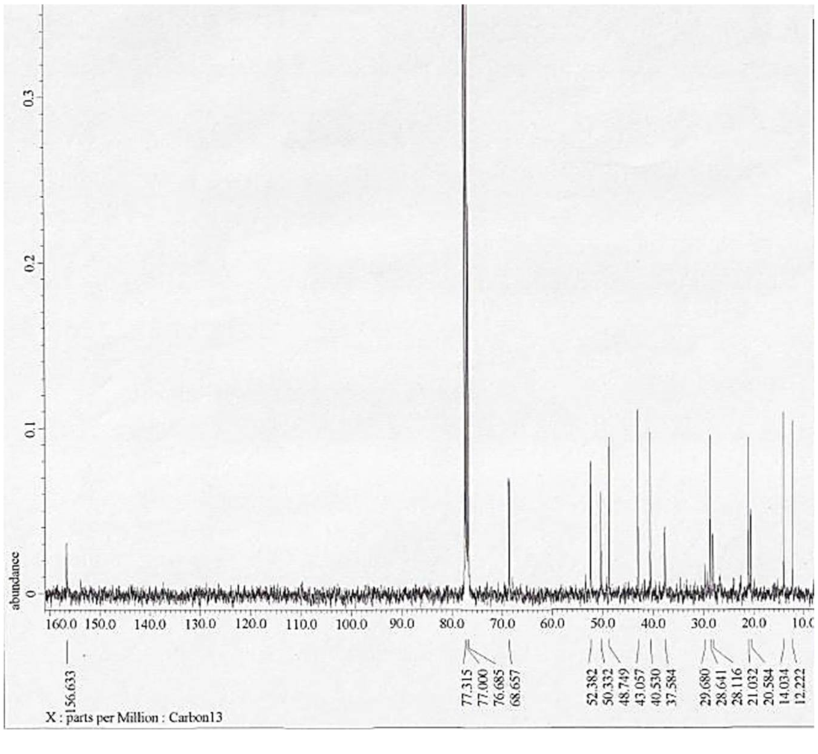
abundance
X : parts per Million : Carbon13
156.633
77.315
77.000
76.685
68.657
52.382
50.332
48.749
43.057
40.530
37.584
29.680
28.641
28.116
21.032
20.584
14.034
12.222

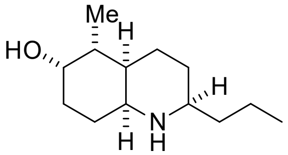
Me
HO
H
H
N
H
H

cis-**211A**

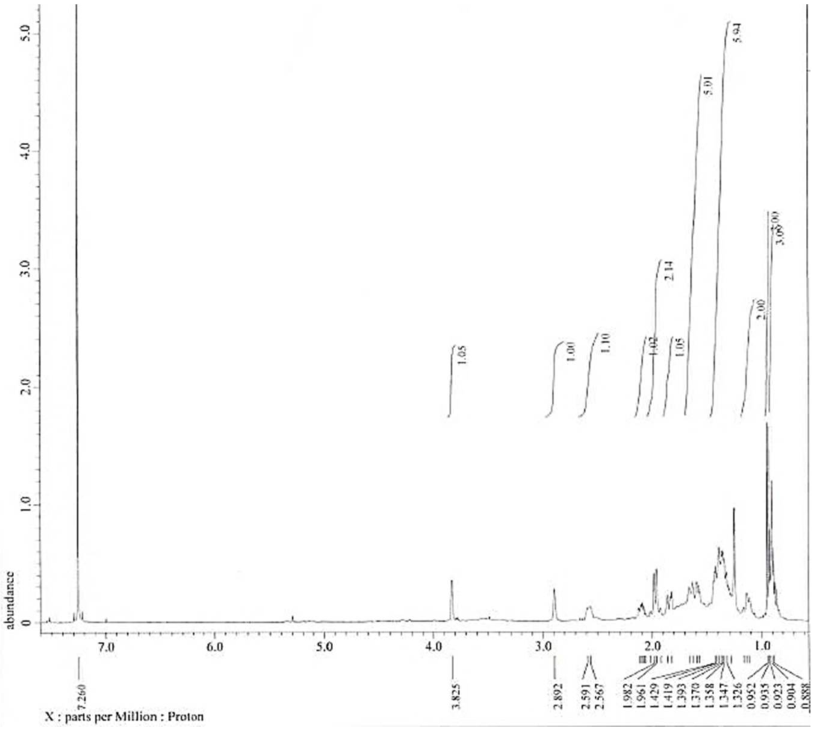
abundance
1.05
1.00
1.10
1.02
2.14
1.05
5.01
5.94
2.00
3.09
3.00
7.260
3.825
2.892
2.591
2.567
1.982
1.961
1.429
1.419
1.393
1.370
1.358
1.347
1.326
0.952
0.935
0.923
0.904
0.888
X : parts per Million : Proton

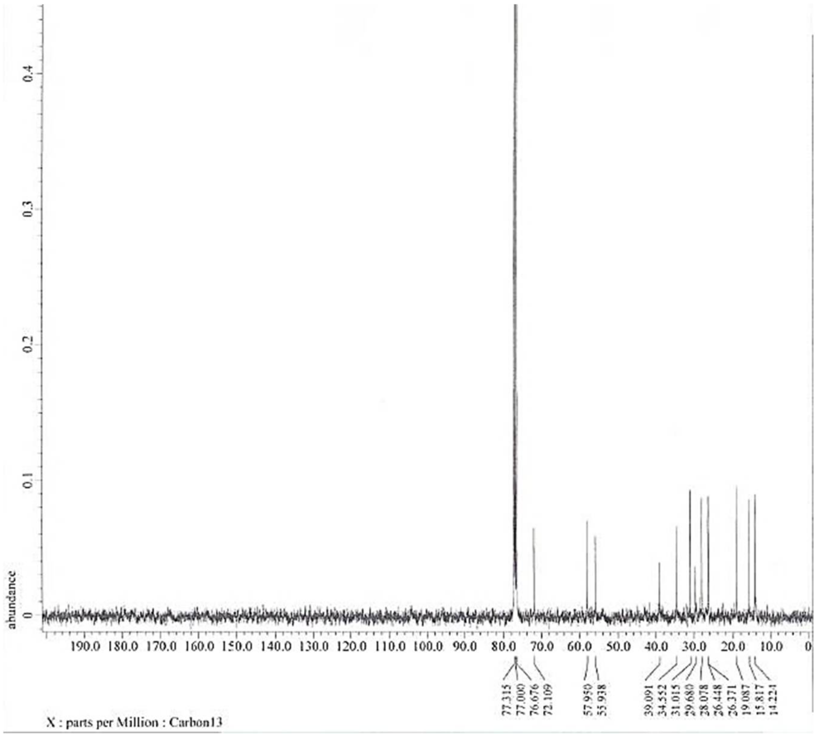
abundance
77.315
77.000
76.676
72.109
57.950
55.938
39.091
34.552
31.015
29.680
28.078
26.448
26.371
19.087
15.817
14.221
X : parts per Million : Carbon13

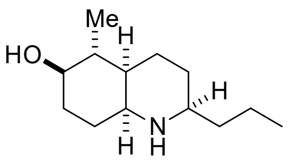
Me
H
HO
H
H
N
H

6-epi-**211A**

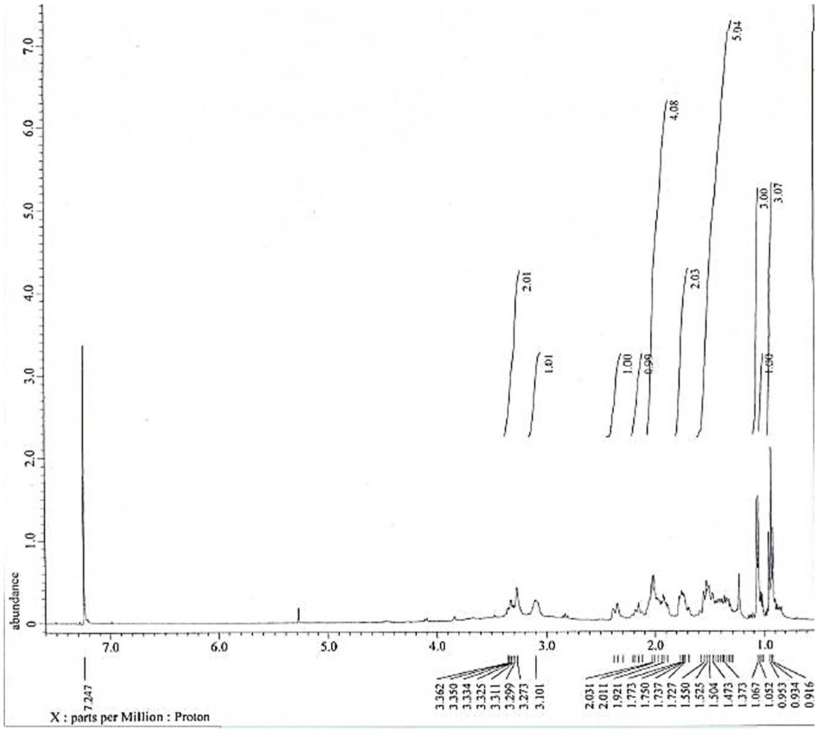
abundance
X : parts per Million : Proton
7.247
3.362
3.350
3.334
3.325
3.311
3.299
3.273
3.101
2.031
2.011
1.921
1.773
1.750
1.737
1.727
1.550
1.525
1.504
1.473
1.373
1.067
1.052
0.953
0.934
0.916
2.01
1.01
1.00
0.99
4.08
2.03
5.94
3.00
3.07
1.00

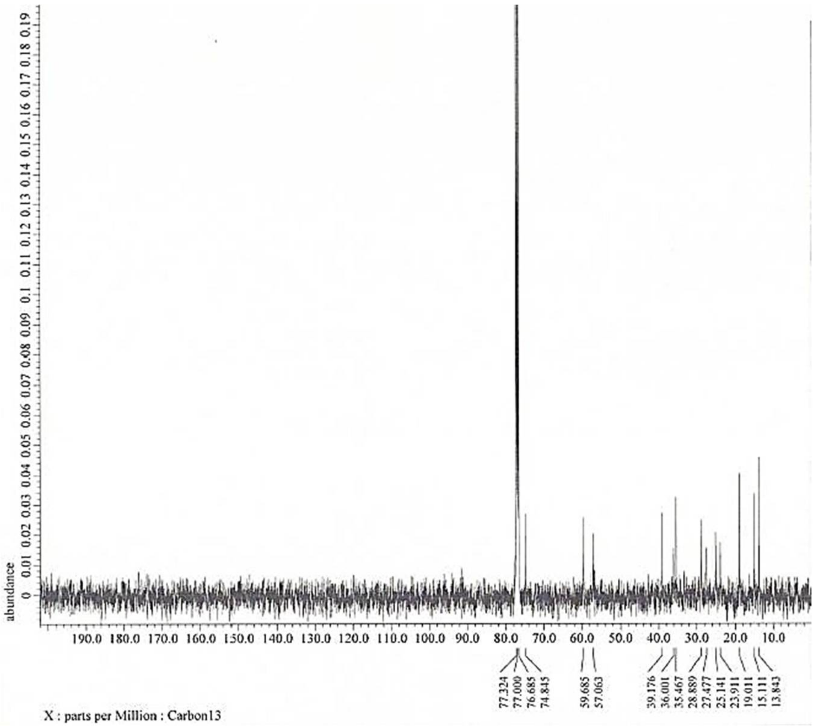
abundance
X : parts per Million : Carbon13
77.324
77.000
76.685
74.845
59.685
57.063
39.176
36.001
35.467
28.889
27.477
25.141
23.911
19.011
15.111
13.843
